# Suicidal chemotaxis in bacteria

**DOI:** 10.1038/s41467-022-35311-4

**Published:** 2022-12-09

**Authors:** Nuno M. Oliveira, James H. R. Wheeler, Cyril Deroy, Sean C. Booth, Edmond J. Walsh, William M. Durham, Kevin R. Foster

**Affiliations:** 1 Department of Biology, University of Oxford, Oxford, UK; 2 Department of Applied Mathematics and Theoretical Physics, University of Cambridge, Cambridge, UK; 3 Department of Veterinary Medicine, University of Cambridge, Cambridge, UK; 4 Department of Biochemistry, University of Oxford, Oxford, UK; 5 Department of Physics and Astronomy, University of Sheffield, Sheffield, UK; 6 Department of Engineering Science, University of Oxford, Oxford, UK

**Keywords:** Antibiotics, Behavioural ecology, Biofilms, Bacterial toxins

## Abstract

Bacteria commonly live in surface-associated communities where steep gradients of antibiotics and other chemical compounds can occur. While many bacterial species move on surfaces, we know surprisingly little about how such antibiotic gradients affect cell motility. Here, we study the behaviour of the opportunistic pathogen *Pseudomonas aeruginosa* in stable spatial gradients of several antibiotics by tracking thousands of cells in microfluidic devices as they form biofilms. Unexpectedly, these experiments reveal that bacteria use pili-based (‘twitching’) motility to navigate towards antibiotics. Our analyses suggest that this behaviour is driven by a general response to the effects of antibiotics on cells. Migrating bacteria reach antibiotic concentrations hundreds of times higher than their minimum inhibitory concentration within hours and remain highly motile. However, isolating cells - using fluid-walled microfluidic devices - reveals that these bacteria are terminal and unable to reproduce. Despite moving towards their death, migrating cells are capable of entering a suicidal program to release bacteriocins that kill other bacteria. This behaviour suggests that the cells are responding to antibiotics as if they come from a competing colony growing nearby, inducing them to invade and attack. As a result, clinical antibiotics have the potential to lure bacteria to their death.

## Abstract (web summary)

Bacteria respond to nutrients and other compounds via chemotaxis, but little is known of their responses to antibiotics. By tracking cells in antibiotic gradients, the authors show that surface-attached *Pseudomonas aeruginosa* move towards antibiotics in what appears to be a suicidal attack strategy.

## Introduction

Bacterial biofilms often experience steep antibiotic gradients when they are treated because the high cell density within these communities both attenuates diffusion and removes compounds from circulation^1–3^. In addition, bacteria commonly live alongside other strains and species that themselves release antimicrobials that diffuse and can again generate chemical gradients^4–6^. In order to cope with life under these conditions, bacteria have evolved a wide variety of physiological responses that detect and respond to toxic compounds. It has been hypothesised that many of these responses evolved as a way to cope with competition from other strains and species (‘competition sensing’^7,8^). For example, bacteria are known to upregulate the production of bacteriocins and antibiotics to generate reciprocal attacks against other toxin-producing strains^9–12^. In addition, there are responses that can defend bacteria from these toxic compounds, such as the upregulation of efflux pumps and biofilm formation^8,13^, which also serve to protect bacteria from clinical antibiotics^14^.

One of the key ways that bacteria physiologically respond to chemicals in their environment is via chemotaxis; the ability of cells to bias their motility in response to chemical gradients^15,16^. There is a large literature on swimming (flagella-driven) chemotaxis, which broadly suggests that cells have the ability to move towards beneficial conditions and away from harmful ones^17–22^. However, to our knowledge, there is no evidence that swimming cells bias their movement in response to antibiotics. Much less is known about the motility responses to chemical gradients of surface-attached bacteria, but the opportunistic pathogen *Pseudomonas aeruginosa* is able to bias its twitching motility across surfaces towards nutrients and other chemoattractants^23–25^. Twitching motility is driven by grappling-hook-like pili that extend and retract to pull bacteria over surfaces and other cells. As such, this form of motility is prevalent within dense communities like biofilms where chemical gradients, including antibiotic gradients, are expected to be most pronounced^1–3^. We hypothesised, therefore, that twitching motility would be important for the ability of cells to survive antibiotics within surface-attached communities like biofilms, which are well known for their ability to tolerate antibiotic treatment^26^. Specifically, we predicted that bacteria would have evolved to move away from antibiotics, thereby enabling a larger population to survive.

Here we show that our prediction was wrong: not only do cells not move away from antibiotics, but we also found that they actively move towards them, killing themselves in the process. Rather than a strategy of moving away from antibiotics, therefore, our work suggests that the cells behave aggressively and respond to antibiotics as though they were coming from competing bacteria.

## Results

### Surface-attached bacteria move towards antibiotics via twitching motility

**Fig. 1:**
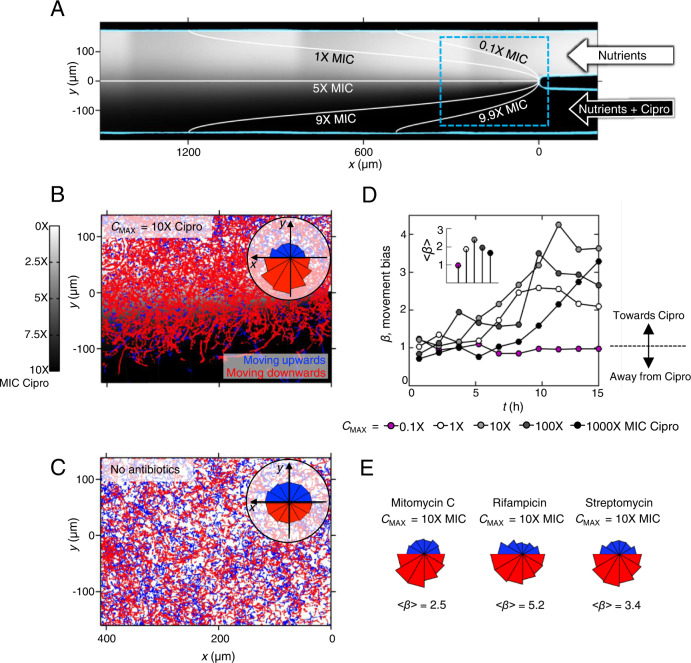
Twitching *P. aeruginosa* cells bias their motility towards increasing antibiotic concentrations. **A** A dual-inlet microfluidic device generates steady antibiotic gradients (e.g. ciprofloxacin, *C*_MAX_ = 10X MIC) via molecular diffusion. Isocontours were calculated using mathematical modelling (Methods) and background shading shows approximate ciprofloxacin distribution visualised using fluorescein. **B** Red (blue) cell trajectories are moving towards (away from) increasing [ciprofloxacin]. Inset: A circular histogram of cell movement direction reveals movement bias towards increasing [ciprofloxacin]. A two-sided binomial test rejects the null hypothesis that trajectories are equally likely to be directed up or down the [ciprofloxacin] gradient (*p* < 0.0001, *n* = 10,714 trajectories). **C** No such bias is seen when nutrient medium without ciprofloxacin is added to both inlets (*p* = 0.854, *n* = 8138). **D** The movement bias, *β* *,* is the number of cells moving up divided by the number moving down the gradient. Cell movement is initially (t < ≈5 h) unbiased (*β* ≈ 1), after which cells bias their movement towards ciprofloxacin (*β* > 1), even when *C*_MAX_ *=* 1000X MIC, (black circles). Movement remains unbiased when *C*_MAX_ = 0.1X MIC, (magenta circles). At *t*  = 15 h, a two-sided binomial test accepts the null hypothesis that trajectories are equally likely to be directed up or down the [ciprofloxacin] gradient for *C*_MAX_ = 0.1X MIC (*p* = 0.811, *n* = 625 cell trajectories) and rejects it for *C*_MAX_ = 1X, 10X, 100X and 1000X (*p* < 0.0001 for each, *n* = 653, 934, 642 and 589 respectively). Inset: The overall bias calculated across all time, <*β* > , shows that *C*_MAX  _= 10X MIC induces the largest response; a two-sided chi*-*squared test that compared the number of trajectories moving up or down the gradient showed that *C*_MAX_ = 10X MIC produced a statistically distinct response from the other *C*_MAX_ values, (*p* < 0.0001, *n* = 13,370 cell trajectories across all data sets). **E** Cells also bias movement towards other antibiotics (*p* < 0.0001, *n* = 1778, 1673 and 3347 trajectories from left-to-right). Figures S2 and S5 show biological repeats and source data are provided as a Source Data file.

We used microfluidic devices and automated cell tracking to quantify the movement of *P. aeruginosa* cells as they are exposed to well-defined spatial gradients of antibiotics in developing biofilms (Fig. 1). We began with the antibiotic ciprofloxacin, which is widely used to treat *P. aeruginosa* infections^27,28^. To set a baseline, we first determined the minimum inhibitory concentration (hereafter MIC) of ciprofloxacin for *P. aeruginosa* (strain PAO1) in shaking cultures, which agrees with the published MIC of this strain (Fig. S1^29^). We then exposed surface-attached cells to an antibiotic gradient in a microfluidic device where the antibiotic concentration ranged from zero to 10 times the MIC (Fig. 1A, B, Methods). After approximately 5 h of unbiased movement, we were surprised to see that twitching cells began to bias their movement towards increasing concentrations of ciprofloxacin (Fig. 1B, D, Movie 1). The movement bias, *β*, defined as the number of cells moving up the gradient divided by the number of cells moving down the gradient, peaks after approximately 10 h and then decays as the surface becomes crowded with cells (Movie 1) and tracking becomes difficult (Methods). The flow through the device also has a small influence on the direction of cell movement because it tends to pull cells in the downstream direction (Fig. 1B, C, E). However, this fluid flow is orthogonal to the direction of the antibiotic gradient, and so does not explain the movement towards antibiotics.

We explored gradients that varied in steepness, always starting at a ciprofloxacin concentration of zero and up to a maximum of 0.1, 1, 10, 100 and 1000 times the MIC, whilst keeping the length scales of the gradient constant. Whenever the maximum concentration was higher than the MIC, cells biased their movement towards the antibiotics (peaking at *β* ≈ 2.5 to 4, Fig. 1D and S2). These experiments revealed that cells are capable of entering regions of extremely high concentration (up to 1000 times MIC) and remain motile for the remainder of the experiments (≈ 5 h; Fig. 1D, S3, Methods). In contrast, the direction of cell movement in a control without antibiotic was approximately random (*β* ≈ 1, Fig. 1C). Importantly, we also see a similar response with other strains of *P. aeruginosa* (PAK and PA14; Fig. S4 and Movie 2).

This motility response is not limited to ciprofloxacin: we found that cells also bias their movement up gradients of antibiotics belonging to different antibiotic classes that have different chemistries and mechanisms of action and do so on a similar timescale to the response seen with ciprofloxacin (Fig. 1E, S5, S6). Ciprofloxacin causes DNA damage by inhibiting an enzyme (gyrase) involved in DNA coiling, but we found that cells also move towards: rifampicin, which targets an RNA polymerase; streptomycin, which targets the ribosome; and mitomycin C, which is again a DNA damaging agent but one that acts by cross-linking DNA. We also tested carbenicillin and other *β*-lactam antibiotics, but these caused extreme cell elongation that inhibited cell movement in our assay and so we did not study them further (Fig. S7). The diversity in both the chemistry and mechanism of action of the antibiotics that elicit biased movement suggests a general response to the cell damage caused by antibiotics, rather than to the specific antibiotics themselves. Indeed, we also find that cells move towards the antimicrobial compound hydrogen peroxide (Fig. S8). Further consistent with the importance of cell damage, we found that sub-MIC concentrations of ciprofloxacin do not bias cell motility (Fig. 1D and S2).

The movement of cells in antibiotic gradients appeared to conform to the definition of chemotaxis: the ability of cells to actively bias their motion in response to chemical gradients^16^. How, though, are cells biasing their motility towards antibiotics? From previous work, we know that cell movement in our assays is driven by twitching motility and that these twitching cells can actively bias their movement towards beneficial nutrients by reversing their movement direction more frequently when moving away from a nutrient source^23^. Is biased motility in antibiotic gradients driven by the same behaviour? To test this hypothesis, we began by quantifying cellular reversal rates and found that cells moving away from ciprofloxacin actively reversed direction more frequently than cells moving towards it (Fig. S9). Our previous study showed that cells lacking the key response regulator of twitching chemotaxis, PilG, remain motile but have significantly reduced reversal rates compared to wild-type (WT) cells and are unable to chemotax towards beneficial nutrients and other chemoattractants^23–25^. Consistent with this previous report, we find here that cells with an in-frame deletion of *pilG* (*ΔpilG*) also have a significantly reduced movement bias towards increasing ciprofloxacin concentrations, despite remaining motile in this assay and having an identical MIC as WT cells (Fig. S1, S10 and Movie 3). As a result, the pilG mutant, unlike the WT, is unable to accumulate in regions containing high ciprofloxacin concentrations (Fig. S11). It is worth noting that the *ΔpilG* cells do show reduced motility in the assay relative to the WT cells but, importantly, our measurements of movement bias only use cells that demonstrate appreciable movement, thus preventing non-motile cells of either genotype from influencing our measurements (Methods). When this *ΔpilG* strain is complemented at the *pilG* locus, its ability to move towards ciprofloxacin is restored (Fig. S10 and Movie 3). Taken together, these results suggest a common behavioural basis for twitching chemotaxis towards antibiotics and nutrients.

### Nutrient gradients do not explain chemotaxis towards antibiotics

Cells only begin to bias their motility up antibiotic gradients after an initial ≈5 h period of nearly random motility (Fig. 1D and S2). This delayed response introduces an important complication, as secondary gradients in nutrients and other compounds potentially released by cells are expected to build up in the device over time, which might indirectly drive the movement towards antibiotics. In particular, cells situated in regions of the device with lower antibiotic concentrations can rapidly proliferate, while cells initially in regions with higher antibiotic concentrations either tend to detach or die (Movie 1). The different numbers of cells on either side of the device, both within the test section (Fig. 1A) and in the regions upstream, means that emergent chemical gradients can form because the cells consume nutrients and release compounds at different rates on either side of the device. Thus, cell movement towards higher antibiotic concentrations could be, in principle, driven by movement towards higher nutrient concentrations and towards lower concentrations of the diverse set of compounds released by cells. We, therefore, sought to confirm whether such secondary gradients could be responsible for the biased movement we observe, rather than it being a direct response to the antibiotic gradients themselves. We first tried switching the direction of the antibiotic gradient, after the cells had established themselves in the regions with lower antibiotic concentrations. However, the sudden change in antibiotic concentration caused cells to either stop moving or detach altogether. We, therefore, sought alternative approaches to control for the formation of secondary de novo gradients.

**Fig. 2:**
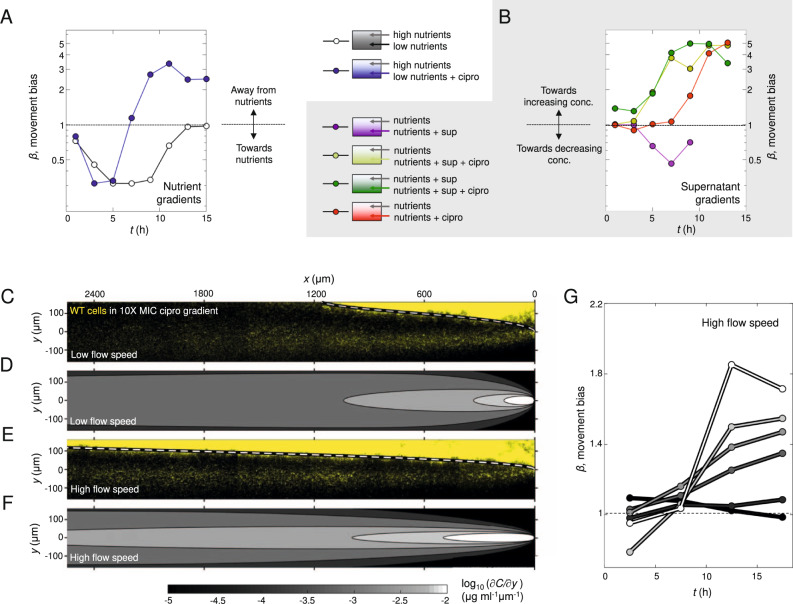
Putative gradients of nutrients or cell products do not explain movement towards antibiotics. **A** Cells move towards increasing [tryptone] (black line,  *C*_MAX_ = 100% of the concentration used in growth medium, *C*_MIN_ = 10%; a two-sided binomial test at *t* = 5 h rejects the null hypothesis that trajectories are equally likely to be directed towards or away from tryptone (*p* < 0.0001, *n* = 132 cell trajectories). However, when ciprofloxacin (*C*_MAX_ = 10X MIC) is added to the lower inlet, cells initially (*t* < ≈7.5 h) move towards increasing [tryptone] (blue line; *p* < 0.0001 at *t* = 5 h, *n* = 110), but then the bias is reversed towards increasing [ciprofloxacin] (*p* < 0.0001 at *t* = 15 h, *n* = 1219). **B** Cells move away from cell-free supernatant (purple line, *C*_MAX_ = 10%; *p* < 0.0001 at *t* = 9 h, *n* = 1464). However, when ciprofloxacin (*C*_MAX_ = 10X MIC) is added to the supernatant, cells rapidly (after ≈ 5 h) move towards increasing [ciprofloxacin] (and thus increasing [supernatant]; light-green line, *p* < 0.0001 at *t* = 13 h, *n* = 1059). Cells similarly move towards ciprofloxacin in a uniform background concentration of 10% cell-free supernatant (dark-green line, *p* < 0.0001 at *t* = 13 h, *n* = 583). **C** After ≈20 h, YFP-labelled WT cells in a ciprofloxacin gradient (*C*_MAX_ = 10X MIC) form dense biofilm at [ciprofloxacin] <1X MIC, whilst a smaller band of migrating cells is visible at much higher concentrations. **D** Coloured regions showing the antibiotic gradient (∂*C/*∂*y*) magnitude using our standard flow speed. **E**, **F** Increasing flow threefold sharpens the gradients, allowing both the 1X MIC isocontour (dashed white line) and the band of migrating cells to stretch further downstream. **G** Movement bias, *β*, increases with ∂*C/*∂*y* (line colours correspond to regions shown in (F)). Least squares linear regression at *t* ≈ 17 h of log_10_(∂*C/*∂*y*) against *β* yielded a slope of 0.258 (95% confidence bounds = 0.191, 0.325). Figures S12 and S17 show biological repeats and source data are provided as a Source Data file.

We focused first on whether putative emergent nutrient gradients could be responsible for the observed biased movement towards antibiotics. Previous work has shown that surface-attached *P. aeruginosa* cells undergo chemotaxis up gradients of the metabolisable carbon source succinate^23^. Consistent with this, we found that twitching cells will also bias their motility up gradients of tryptone, the growth medium used in this study (Fig. 2A and Fig. S12), a response that has also been observed in swimming *P. aeruginosa* cells^30^. To test whether the biased movement we see in our experiments with antibiotics could be driven solely by de novo gradients in tryptone, we created opposing gradients of tryptone and ciprofloxacin in our microfluidic device. Specifically, we injected full strength tryptone through one inlet of the device and ciprofloxacin mixed with tryptone at 10% of the regular concentration through the other inlet. We use 10% media rather than 0% because, with the latter, we find that cells quickly stop moving in regions without nutrients. If cells in our previous experiments are simply moving in response to nutrient gradients, we expect that cells would now move away from the antibiotic source. While cells initially move in the direction of increased tryptone, after ≈5 h this response rapidly drops off and after ≈7.5 h, cells again exhibit biased movement in the opposite direction towards increasing antibiotic concentrations (Fig. 2A and Fig. S12). The robust movement of cells towards low nutrient and high antibiotic concentrations so early in the experiment suggests that chemotaxis towards antibiotics (Fig. 1) is not driven by nutrient gradients.

### Repulsion by cell-free supernatant does not explain chemotaxis towards antibiotics

We next explored the possibility that biased movement in antibiotic gradients could be explained by products released by cells that might trigger repulsion away from regions of the device with high cell density. Consistent with the potential for repulsion from areas of high cell density, we find that twitching cells are repelled by cell-free supernatant extracted from cells grown to high density under static conditions in the multi-well plate assays that are widely used to study biofilm physiology (Fig. 2B, S12, Methods^13,31^). Specifically, we mixed cell-free supernatant collected from static well-plates with fresh media (20% supernatant, 80% tryptone broth) and injected it through one inlet of our device, whilst media mixed with the same proportion of water (20% water, 80% tryptone broth) was injected through the other inlet. These experiments showed that cells move away from the channel containing supernatant (Fig. 2B and Fig. S12, purple lines). This result is interesting as it suggests that in *P. aeruginosa*, twitching motility will guide cells away from regions of high cell density in developing biofilms, potentially facilitating the colonisation of new territory. However, it also raises the possibility that this repulsion could explain the migration towards antibiotics. We, therefore, performed a number of additional experiments to explore this possibility.

We reasoned that if a factor released by cells is responsible for movement towards antibiotics, then adding cell-free supernatant in the background of an antibiotic gradient should limit or stop the movement of cells toward higher antibiotic concentrations. We tested this hypothesis with two additional experiments. The first experiment exposed cells to a gradient of ciprofloxacin in a uniform background of cell-free supernatant. We again used cell-free supernatant collected from high-density static cultures at 20% concentration, which we expected to saturate any gradients in cell products that might occur de novo in the microfluidic device where cell densities are initially low (Fig. 2B and Fig. S12, green lines). The second experiment exposed cells to a ciprofloxacin gradient and supernatant gradient simultaneously, with the larger concentration of both on the same side of the device, so that repulsion of cells away from supernatant would oppose the putative movement towards ciprofloxacin (Fig. 2B and Fig. S12, yellow lines). We still observed movement towards ciprofloxacin in both of these experiments. In fact, we found that movement towards ciprofloxacin actually commences more rapidly in the presence of the cell-free supernatant than without (Fig. 2B and Fig. S12, red lines). This more rapid response could occur because the cell-free supernatant helps the cells to tolerate antibiotics or because a factor in the supernatant promotes the chemotaxis response itself. Either way, it shows that the delay before bacteria begin the chemotaxis response is labile and can be greatly reduced under some conditions (see also Fig. S4). Importantly, this rapid migration towards antibiotics occurred even when the cell-free supernatant gradient was oriented such that one would expect it to repel cells away from the antibiotics. In sum, repulsion due to factors present in cell-free supernatant can promote cell movement from regions of high cell density, but this effect does not explain the observed chemotaxis towards ciprofloxacin.

There remains the possibility that chemotaxis towards antibiotics is driven by a repulsive factor, or set of factors, that are not present in the cell-free supernatant used in the above experiments, which was collected from static cultures grown in multi-well plates. For example, it may be that cells growing under flow in the microfluidic device have different physiology, such that any secondary chemical gradients that develop within the device are not captured by adding cell-free supernatant collected from static cultures on the bench. We, therefore, repeated the cell-free supernatant experiments using media collected directly from cells growing within microfluidic devices (Fig. S13, Movie 4, Methods). To maximise the likelihood of observing a response, we used 100% cell-free supernatant for these experiments and flowed this through one inlet, with growth media (tryptone broth) through the other. This revealed that, as for the standing culture experiments, cells move away from the channel with cell-free supernatant obtained from microfluidic devices. With 100% cell-free supernatant, this response is likely to be a combination of both moving away from cell-free supernatant and towards an increasing nutrient concentration. When we combined the cell-free supernatant with 10X MIC ciprofloxacin, cell movement was initially biased away from the cell-free supernatant/ ciprofloxacin. However, importantly, after ≈7 h, cells began to move in the opposite direction, towards the cell-free supernatant and ciprofloxacin. The addition of cell-free supernatant on the antibiotic side of the device, therefore, does not prevent chemotaxis towards the antibiotic, as would be expected if cell products released in the device were driving the response. These experiments again suggest that repulsion by cell-free supernatant is not sufficient to explain migration towards antibiotics.

### A factor released in response to antibiotics does not explain chemotaxis

An additional possibility is that chemotaxis is driven by a repulsive factor that is not present in the cell-free supernatants we studied. These supernatants were collected from experiments without antibiotic present, as any residual antibiotic remaining in the collected supernatant would complicate interpretation. However, this leaves open the possibility that surface-attached cells within our microfluidic experiments might release a compound in response to the antibiotics (i.e. antibiotic-induced cell products) that could drive directional cell movement, something that has been observed for swarming bacteria^32^. To examine this possibility, we used a classical result from the chemotaxis literature: movement bias is predicted to increase with the strength of the chemical gradient. This correlation has not only been demonstrated in chemotaxis occurring in twitching bacteria^23^, but also in swimming bacteria^15^ and eukaryotic cells^33^. Since the distribution of ciprofloxacin in our microfluidic devices is predicted to differ starkly from that of putative antibiotic-induced cell products (Figs. S14, S15), measuring how movement bias changes at different positions within the device gives us the ability to distinguish which of these alternatives is most likely to explain the observed patterns of cell movement.

In order to do this, we first wanted to estimate how the concentration of ciprofloxacin varies in space in the device. Here, a model of diffusion was used to quantify how the concentration of ciprofloxacin, and its spatial gradient, varies within our device (Methods). Using a diffusion coefficient of *D* = 200 µm^2^ s^−1^, the isocontour corresponding to the MIC of our strain (1X MIC) was found to closely match the position where biofilm growth becomes strongly suppressed by antibiotics (dashed line, Fig. 2C, E). If the mean flow speed within the device is increased by a factor of three (from 42.3 µm sec^−1^ used as our baseline, to 127 µm sec^−1^, see Methods), the 1X MIC isocontour was predicted to be pushed further downstream, which again closely matches the distribution of biofilm experimentally observed under this higher flow condition (Fig. 2E), suggesting our model is working as expected. While the diffusion coefficient of ciprofloxacin is not as well-known as some other compounds, our fitted value of *D* (where the 1X MIC isocontour follows the line where biofilm growth is suppressed) is within a factor of two of previous estimates for ciprofloxacin^34–36^. We also tested how different values of *D* would affect the distribution of antibiotics in our microfluidic device (Fig. S16). These analyses indicate that the lower boundary of the thick biofilm in our device might occur at a slightly higher or lower concentration compared with the MIC measured in shaking liquid culture^6^. However, the general features of the distribution of antibiotics in our device are not sensitive to the precise value of *D*. In particular, no matter what value of *D* is used, the concentration is always half the maximum along the centreline and the steepest gradients always occur along the centreline. In the subsequent analyses, we assumed *D* = 200 µm^2^ s^−1^. These experiments also reveal that within the region close to the centreline (where the gradient is steepest), cell movement is highly biased towards antibiotics (Fig. 2C–G). This process can be observed dynamically in the inset of Movie 1, where cells along the centreline of the device move between a region of high cell density on the antibiotic-free side of the channel and a second region of high cell density that forms as cells begin to accumulate in high antibiotic concentrations.

Next, we calculated the movement bias, *β*, as a function of both time and the local ciprofloxacin gradient (∂*C/*∂*y*). These analyses used a high flow speed (mean speed = 127 µm sec^−1^) so that chemotaxis could be observed further downstream in the device (Fig. 2E, F). This allowed us to simultaneously image cells in four different fields of view so that we had a sufficient number of cell trajectories in each bin (Fig. 2E, F, Methods). Our analyses reveal that the strength of the ciprofloxacin gradient (∂*C/*∂*y*) is an excellent predictor of *β* – after an initial period of random motility, cells experiencing a larger ∂*C/*∂*y* were observed to have a larger *β* (Fig. 2G and Fig. S17). These results indicate that the stimulus the cells are responding to closely matches the predicted gradient of ciprofloxacin within our device. While the gradient of ciprofloxacin decreases as one moves downstream, the gradient of a putative antibiotic-induced cell product would likely increase in the downstream direction. This is because as fluid passes through the device, it passes by more and more cells exposed to sub-MIC levels of antibiotics, which would steadily increase the concentration of the product on one side of the device (Figs. S14, S15). While the exact distribution of a putative cell product is hard to predict, the fact that it would differ strongly from that of ciprofloxacin suggests that the excellent correlation between ∂*C/*∂*y* and *β* (Fig. 2G and Fig. S17) is unlikely to be driven by antibiotic-induced cell products.

Taken together, our data suggest that neither nutrient depletion, nor products released either in the presence or absence of antibiotics, can explain the movement of cells towards ciprofloxacin. These results suggest that we are observing a direct response to the antibiotics. There is a growing body of work showing that flagella-based swimming can transport bacteria into regions of high antibiotic concentration^37–40^. However, to the best of our knowledge, there is no evidence of chemotaxis in response to the antibiotic gradients themselves. Here, we observe that cells move towards antibiotics, actively reverse direction to facilitate this directed movement and, finally, that movement bias increases with the strength of the antibiotic gradient, which are all signatures of chemotaxis^15,16^. As discussed above, this chemotaxis occurs towards a range of antibiotics (and hydrogen peroxide) that have different mechanisms of action, which suggests that the biased migration is driven by a general response to the effects of antibiotics on cells.

### Cells migrate towards antibiotics and die

Our data show that *P. aeruginosa* cells will bias their motility to move into regions with extremely high antibiotic concentrations. Moreover, many cells remain motile at concentrations many times greater than that needed to prevent their growth in a standard liquid culture assay (Fig. S3). This ability to persist in the presence of antibiotics is likely to be enabled by stress responses, such as the SOS response to DNA damage in the case of ciprofloxacin. This response stalls cell division while cells attempt to repair DNA damage^41^. We wanted to establish the ultimate fate of the migrated cells: do they remain viable and gain tolerance to high concentrations of antibiotics, or are they terminal and doomed to die? Or indeed, is it possible that some cells evolve mutations that confer resistance in the short time-scale (≈20 h) of our experiments^37,39^? The challenge with answering this question is that the cells in question are fully encased within PDMS-based microfluidic devices, which means it is not possible to isolate migrating cells and study them further. One can watch them for the duration of the experiment, but after ≈20 h, the devices become clogged with growing cells. We are, therefore, unable to ascertain whether cells that migrate into regions of high antibiotic concentration remain active and retain long-term viability.

**Fig. 3:**
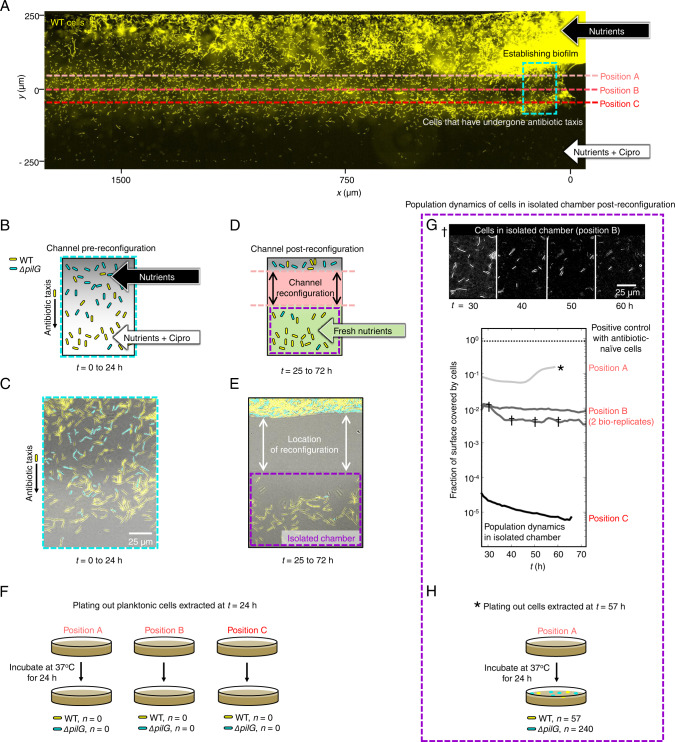
Cells migrate towards antibiotics and die. **A** YFP-labelled WT cells (yellow) and unlabelled, chemotaxis-impaired mutant cells (*ΔpilG*, not visible in this epi-fluorescent image) were exposed to ciprofloxacin gradients (*C*_MAX_ = 100X MIC) in fluid-walled microfluidic devices. At *t* ≈ 24 h, channels were reconfigured (dashed red lines) in separate experiments at either ‘Position A’, isolating all chemotaxing cells, or ‘Position B’ or ‘C’, which were progressively more conservative and isolated cells that had migrated further towards *C*_MAX_. Cartoon **B** and experimental image **C** corresponding to the approximate location of the dashed-cyan box in (**A**) immediately prior to channel reconfiguration. We predicted – and experimentally confirmed – that the WT (yellow), but not *ΔpilG* (cyan), would move towards ciprofloxacin. Image representative of four independent experiments. **D** Channel reconfiguration aimed to isolate chemotaxing WT cells in an isolated microfluidic chamber containing antibiotic-free nutrient medium. **E** Experimental image of the cells after channel reconfiguration confirms formation of an isolated fluid chamber with mostly WT cells (mean proportion of WT over nine independent samples = 84% with 95% confidence intervals of 81-87%; a two-sided Z-test rejected the null hypothesis of 50% WT cells, *p* < 0.0001, *n* = 5074). **F** Planktonic cells extracted from the chamber and monitored for growth on nutrient agar showed no detectable growth. **G** Chambers were imaged for ≈40 h to monitor any remaining cells. No cell growth was detectable when channels were reconfigured at Positions B and C and the chamber surfaces gradually cleared as cells died or detached (see also the image series marked by †, which is representative of the three separate experiments). When reconfigured at Position A, the fraction of the chamber surface covered by cells initially decreased, momentarily increased, and finally plateaued at ≈10% coverage. After *t* ≈ 60 h (see*), we extracted the entire chamber contents and monitored growth on agar plates lacking antibiotics. **H** After a further 24 h, we recovered a small number of both WT and *ΔpilG* colonies in the Position A experiment, suggesting we had isolated some cells that had not yet reached lethal antibiotic concentrations. Source data are provided as a Source Data file.

In order to overcome this challenge, we moved to a novel assay that uses open “fluid-walled” microfluidics^42^, where twitching cells can be studied in a chemical gradient and then isolated after they have migrated, (Methods; for a detailed description of our approach, see^43^). Briefly, microfluidic devices (Fig. S18) were inoculated with a 1:1 co-culture of YFP-labelled WT cells and unlabelled, chemotaxis-null *ΔpilG* cells, which allowed us to follow cells that can and cannot perform chemotaxis. We then followed cell movement under the microscope until a substantial number of WT cells had begun to migrate towards ciprofloxacin (*C*_MAX_ = 100X MIC, *t* = 24 h; Fig. 3A–C). To reconfigure the fluid-walled channels, we mounted a PTFE (polytetrafluoroethylene) stylus^44^ to the microscope’s condenser and moved the device relative to the stylus using the microscope’s motorised stage. This technique allowed us to reconfigure the central channel into segments in real-time and with a high level of accuracy (tens of micrometres). Thus, we could physically isolate the cells that had performed chemotaxis within a fluid-walled chamber (Fig. 3D, E). Importantly, these cells were strongly biased towards WT rather than the chemotaxis-impaired *ΔpilG* cells (mean = 84% WT, Fig. 3E), confirming our ability to isolate cells that could undergo chemotaxis. To analyse the viability of these isolated cells, we extracted the contents of the chambers and plated them onto agar plates without antibiotics to monitor whether any of the cells were able to form viable colonies. Additionally, we replaced the chamber contents with fresh growth media without antibiotics to monitor the health of any cells that remained attached to the surface of the microfluidic chambers.

We reasoned that cell viability might depend on how far a cell has migrated up the ciprofloxacin gradient and, thus the ciprofloxacin concentration it has experienced. Therefore, we carried out three separate experiments in which the microfluidic channels were reconfigured at different positions, allowing us to isolate three different sub-populations of cells that had each experienced a different minimum concentration of ciprofloxacin. In the first position, we reconfigured the channel to isolate only those cells that had already undergone chemotaxis and thus begun to accumulate in regions of high ciprofloxacin concentration (“Position C”, Fig. 3A). The cells extracted from this sub-population were found to be incapable of forming colonies on antibiotic-free agar (Fig. 3F), and none of the cells that remained within the isolated fluid chamber underwent any further cell division in over 36 h (Fig. 3G). To confirm that the fluid chamber provided a suitable environment for cell growth, we inoculated ≈25 healthy cells into the chamber at the end of the experiment. These freshly-inoculated cells grew rapidly, completely covering the bottom surface of the chamber within 24 h (dotted line, Fig. 3G), demonstrating that the chamber did not contain residual antibiotics that could stifle growth.

We next reconfigured the channel to isolate both cells that had already undergone chemotaxis as well as the majority of cells that were still migrating (“Position B”, Fig. 3A). Once again, none of these cells showed evidence of long-term viability (Fig. 3F, G). Finally, we reconfigured the channel to isolate all cells that were undergoing or had already undergone chemotaxis, including those that had only very recently begun to start moving up the ciprofloxacin gradient (“Position A”, Fig. 3A). Once again, the extracted cells were found to be incapable of forming colonies (Fig. 3F). Whilst there was initially no detectable cell growth within the fluid chamber, after ~20 h, the early stages of a biofilm containing both WT and *ΔpilG* cells was observed forming on the surfaces of the fluid chamber (Fig. 3G). After a further 10 h, the growth of this biofilm appeared to have stalled and so the entire chamber contents were extracted and plated onto antibiotic-free nutrient-rich agar plates, on which a relatively small number of colonies were recovered (Fig. 3H). Interestingly, while the majority of cells seen to migrate towards antibiotics were WT, the majority of colonies that grew were chemotaxis-impaired *ΔpilG* cells, which were less likely to enter the regions of high antibiotic concentration. Taken together, these three separate channel reconfigurations suggest that whilst some cells retain long-term viability at the earliest stages of chemotaxis, they will continue to move into higher antibiotic concentrations where they rapidly lose this viability and will ultimately die.

### Twitching bacteria move towards supernatant from competitors

Why does *P. aeruginosa* perform an ultimately fatal migration towards antibiotics? We hypothesized that the observed migration towards clinical antibiotics may have its evolutionary origins in the natural ecology of *P. aeruginosa*. Clinical antibiotics are widely reported to induce biofilm formation and this can be recapitulated in *P. aeruginosa* using the cell-free supernatant of other *P. aeruginosa* strains^13^. However, this effect is only observed when the supernatant is toxic to the focal strain, which is consistent with the hypothesis of competition sensing: an evolved ability to detect and respond to harmful competing strains^7^. Under this hypothesis, the presence of clinical antibiotics causes bacteria to act as though the attack is coming from ecological competitors^13^.

**Fig. 4:**
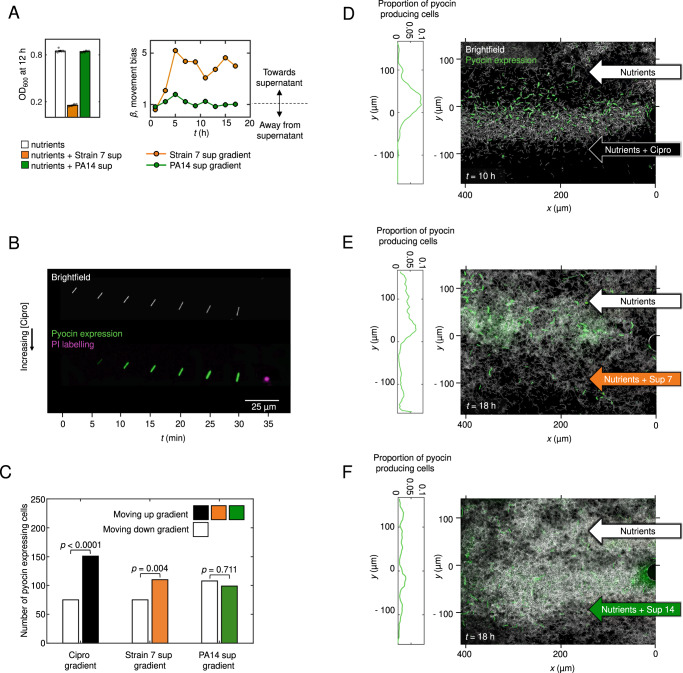
Cells produce bacteriocins (pyocins) as they move towards both clinical antibiotics and toxic supernatant collected from a competitor strain. **A** Our focal PAO1 strain is inhibited by cell-free supernatant of the soil-isolate Strain 7 but not of the clinical isolate PA14 (grey circles show measurements from 8 independent replicates; error bars show standard error). PAO1 moves towards the toxic supernatant from Strain 7 (*β* > 1, orange line; a two-sided binomial test at *t* = 17 h rejects the null hypothesis that trajectories are equally likely to be directed towards or away from supernatant (*p* < 0.0001, *n* = 911 cell trajectories)), but not towards the supernatant from the non-inhibitory PA14 (*β* ≈ 1, dark-green line*; p* = 0.669 at *t* = 17 h, *n* = 1333). Biological repeat shown in Fig. S19. **B** A representative PAO1 cell (see also Movie 5) undergoing chemotaxis towards ciprofloxacin gradually turns on pyocin expression (as visualised with an mNeonGreen reporter). The cell eventually undergoes lysis seen as a transient burst of DNA-labelling propidium iodine (shown in purple at *t* = 35 min). **C** Pyocin-expressing cells are more likely to move towards higher concentrations of ciprofloxacin and toxic Strain 7 supernatant (a two-sided binomial test rejects the null hypothesis that cells were equally likely to be moving towards or away from chemoattractant with p-values of *p* < 0.0001 and *p* = 0.004 respectively). This bias is absent in gradients of non-toxic PA14 supernatant (*p* = 0.711). Counts are pooled across two bio-replicates. **D** After 10 h in a ciprofloxacin gradient (*C*_MAX_ = 10X MIC), many cells have undergone chemotaxis towards increasing ciprofloxacin concentrations and often express pyocins as they do so (quantified using mNeonGreen fusion). The proportion of cells expressing pyocins peaks near the middle of the microfluidic channel, where the ciprofloxacin gradient is relatively steep. **E** Similarly, many cells chemotaxing towards toxic Strain 7 supernatant also express pyocins. **F** Pyocin expression is also seen in gradients of non-toxic PA14 supernatant but cells do not bias their movement here. Source data are provided as a Source Data file.

We, therefore, asked whether cells might bias their movement up gradients of toxic cell-free supernatant isolated from different *P. aeruginosa* strains, in a similar way to their response to clinical antibiotics. Based on previous work, we selected one strain (‘Strain 7’) that strongly inhibits the growth of our focal strain PAO1 in coculture and a second strain (PA14) that does not (Fig. 4A^13^). Consistent with our hypothesis, we observed biased movement towards the toxic supernatant that stifles cell growth, but unbiased movement in a gradient of supernatant from the non-toxic strain (both using a supernatant concentration of 10%, Fig. 4A and Fig. S19). These data again suggest that the response we observe with clinical antibiotics is a general response to a gradient of toxic components. But why would cells move towards toxic compounds? Moving towards toxic stimuli may be a maladaptive response driven by their adverse effects on cells. Alternatively, it may constitute part of a general strategy to attack and invade the territory of neighbouring genotypes that present a threat. Consistent with the latter, the evolution of mass suicide to release toxins was recently documented in non-motile *Escherichia coli*^9^. Such aggressive strategies also have a precedent in the animal world, particularly in the social insects that are well known for territorial behaviours, where workers will actively seek out and attack neighbouring colonies and other harmful species, and die in the process^45^.

In this model, *P. aeruginosa* has evolved to move towards competitors in order to engage in a counter-attack. If this model is correct, we reasoned one should see the release of antimicrobials by *P. aeruginosa* as it migrates up the gradient of antibiotics or toxic cell-free supernatant. In order to test this hypothesis, we built a fluorescent transcriptional reporter for the Pyocin R2 of *P. aeruginosa*, which is induced by DNA damage and released via programmed cell suicide that causes cell lysis^46^. We observed this strain in gradients of ciprofloxacin and either the toxic, or non-toxic, cell-free supernatant of another *P. aeruginosa* strain. These experiments confirmed that many cells expressed pyocins and often performed explosive lysis, which is known to release pyocins (Fig. 4B–F, see also^47^). These lysis events can be seen clearly in the presence of propidium iodine which stains the free DNA released upon lysis, observed as a transient puff of colour (Fig. 4B, Movie 5). When combined with chemotaxis, the result is the biased movement of pyocin-releasing cells towards the source of toxins (Fig. 4C). The patterns of pyocin release are particularly striking in the case of ciprofloxacin (Fig. 4D, Movie 5). This observation fits with the known regulation of pyocins, which is DNA damage dependent^29,46^, and suggests that DNA-damaging agents (like ciprofloxacin) will lead to a particularly aggressive response. Consistent with this, the release of colicin bacteriocins in *E. coli* is also known to be upregulated by DNA-damaging toxins, albeit in stationary cells^9^. Altogether, our experiments suggest that *P. aeruginosa* has evolved to bias its twitching motility towards harmful compounds while releasing bacteriocins, which is consistent with a counter-attack behaviour.

## Discussion

Our experiments reveal that the pathogen *P. aeruginosa* responds to spatial gradients of antibiotics in a surprising way. Rather than moving away from toxins, as we initially hypothesised would happen, cells actively bias their motion to undergo chemotaxis towards lethal concentrations of antibiotics. We observe this response for a range of different clinical antibiotics and for inhibitory supernatant from foreign *P. aeruginosa* cells. This finding suggests that the biased movement is driven by a general effect of the cell damage caused by antibiotics, rather than a specific response to the compounds themselves. The weight of our evidence also suggests that cells are responding directly to toxin gradients but, until the mechanistic basis of the response is understood, there will remain the possibility that a secondary effect of the antibiotics is driving the cell migration patterns that we observe. However, the effects of chemotaxis are clear either way - in the presence of a spatial gradient of toxins, cells change their behaviour, move towards increasing concentrations, and undergo bacteriocin production. In the context of our experiments, this behaviour is also lethal for the cells that reach high concentrations of antibiotics; we observe suicidal chemotaxis. However, we note that in other contexts, it is possible that some cells would survive the migration, such as when an incoming toxin is on the wane.

The behaviour we observe is consistent with a robust response of *P. aeruginosa* to the threat of ecological competition from other bacteria. *P. aeruginosa* is already known to be a strong retaliator from its use of the type VI secretion system, which it activates multiple times in response to a single incoming attack from a competitor^48,49^. It is also notable as a species that carries a particularly large number of antibacterial weapons^45^. A growing body of evidence, therefore, suggests that *P. aeruginosa* is a highly aggressive species that will attempt to eliminate competitive strains and species growing in its neighbourhood. However, in the context of clinical antibiotics, this strategy works against this species, as the drugs simply lure the cells to their death.

## Methods

### Strains, media and culture conditions

The main strain used throughout this work is a WT *P. aeruginosa* PAO1 from the Kolter collection, ZK2019^50^. In Fig. S4, we also use *P. aeruginosa* PA14 (ZK2466, from the Kolter collection) and *P. aeruginosa* PAK (van Delden lab). The *P. aeruginosa* strains used as competitors (Fig. 4), are also from the Kolter collection and have been published and described elsewhere^13^. They include the clinical isolate PA14 (ZK2466) and the environmental isolate ‘Strain 7’ (ZK3098^13^), which notably is distinct from the strain PA7 used in other studies^51^. To study the role of cellular reversals in driving biased movement towards antibiotics (Figs. S9–11), we used strains with an in-frame deletion mutation in *pilG* (*ΔpilG)* and a strain complemented at the *pilG* locus, both of which have been published and described previously^52^. For co-culture experiments and to confirm that strain background was not an issue (Fig. 3), we also generated an in-frame *pilG* deletion in our (Kolter) PAO1 WT strain background using allelic replacement vector pJB118 published and described previously^52^. This strain was used in co-culture experiments with a YFP-labelled variant of our PAO1 WT, which has also been published and described elsewhere^13^. To study pyocin expression (Fig. 4), the mNeonGreen^53^ gene, with a ribosome binding site (CTAGATTTAAGAAGGAGATATACAT) was inserted immediately after the stop codon of PA0631 (the final gene of the pyocin R2 operon) by two-step allelic exchange. A construct including the mNeonGreen gene flanked by ≈400 bp up and downstream of the insertion site was synthesized (IDT) and cloned into the MCS of pEXG2^54^ using NEB Hifi assembly (NEB). This construct was introduced into our (Kolter) PAO1 WT mentioned above by standard methods^55^ and confirmed by sequencing using primers flanking the insertion site (GCGGTGCTGTCATGAGCC/ GGATGTCACCTGCAACCTCA).

All bacterial cultures were grown the day before experiments in shaken LB media at 37 **°**C. On the day of the experiment, these were sub-cultured to obtain cells in the exponential phase. These were then diluted in tryptone broth (TB, 10 g Bacto tryptone per 1 L water), the growth medium used in all of our microfluidic-based experiments. To find the minimum concentration of each antibiotic that inhibits growth in homogeneous cultures, we used the same growth medium but supplemented with different antibiotic concentrations. Exponential phase cells from overnight cultures were inoculated in flat-bottomed 96-well plates (Nunc) in 200 μl of TB at a starting optical density (at 600 nm) of 0.05, and then were placed inside a plate reader (Tecan, Infinite M200 Pro) for 20 h, where they grew at 22 °C under shaken conditions. Measurements were recorded every 30 min and the MIC was defined as the minimum concentration of antibiotic that prevents detectable growth after 20 h (Fig. S1).

### Microfluidic experiments

All microfluidic experiments were imaged using a Zeiss Axio Observer inverted microscope with Zeiss software (Zen Blue, 2012), a Zeiss 20X Plan Apochromat objective, Zeiss AxioCam MRm camera, a Zeiss HXP 120 light source, a Zeiss Definite Focus system, and Zeiss filter sets (38 HE, 46 HE, and 63HE) except for the experiment shown in Fig. S13, which was imaged using a Nikon Ti-E inverted microscope with a Hamamatsu Flash 4.0 v2 camera, Nikon software (NIS-Elements AR v4.51.01), Nikon Perfect Focus system and a Nikon 20X Plan Apochromat objective. We maintained the temperature at 20–22 **°**C, but when our microscope suite was outside of this range, we used a custom-designed microscope incubation chamber with both heating and cooling modes (Digital Pixel Cell Viability and Microscopy Solutions, Science Park Square, Brighton, UK; http://www.digitalpixel.co.uk) to maintain a constant temperature.

We used the BioFlux 200 system (Fluxion Biosciences) for all microfluidic experiments, with the exception of the microfluidic experiments shown in Fig. 3 (see below). When using the BioFlux 200 system, we followed closely our previously published experimental protocol^23^. Exponential phase cells were introduced in dual-inlet glass bottomed microfluidic channels (24-well plate, product-number 910-0050) and allowed to attach to the surface in the absence of flow for 10-15 min. After flushing out the planktonic and weakly adhered cells from the test section, we kept the flow rate constant at 4 µl h^−1^, (or 12 µl h^−1^ in the high flow speed experiments, Fig. 2E–G and Fig. S17), and imaged cells using brightfield microscopy at a rate of 1 frame min^−1^. The flow rate of 4 µl h^−1^ corresponds to a mean flow speed of 42.3 µm sec^−1^ and a wall shear stress of 0.04 dyn cm^−2^, whilst the flow rate of 12 µl h^−1^ corresponds to a mean flow speed of 127 µm sec^−1^ and a wall shear stress of 0.13 dyn cm^−2^. Stable antibiotic gradients were generated in the test section of the microfluidic channels via molecular diffusion by injecting growth medium through both inlets and the test antibiotic through just one of them (Fig. 1).

To estimate the distribution of antibiotic in the dual-inlet BioFlux microfluidic experiments (Fig. 1A), we flowed fluorescein through one inlet and buffer through the other. This fluorescent dye was imaged using the aforementioned Zeiss microscope with scanning laser confocal attachment (Zeiss LSM 700), a Zeiss EC Plan Neofluar 10X objective, a Zeiss LSM T-PMT unit and Zeiss software (Zen Black, 2012) to simultaneously visualise the channel geometry. We recorded z-stacks of images at multiple, overlapping positions along the length of the device and the maximum fluorescent intensity in *z* was used to obtain the relative fluorescein concentration at each *x, y* position (Fig. 1A). To simulate the distribution of antibiotics in our BioFlux device (Fig. 1A, 2C–F), we developed a mathematical model that incorporates the combined effect of fluid advection and molecular diffusion (see section below for details).

We used epi-fluorescent microscopy to visualise propidium iodide and the mNeonGreen fluorescent protein of our pyocin reporter strain (Fig. 4B–F). Propidium iodide (30 µM) was added to the growth medium that was injected through both inlets of our devices. Epi-fluorescent images were recorded at a frame rate of 0.1 frame min^−1^ with exposure times of 90 and 200 ms for the propidium iodide and mNeonGreen pyocin reporter, respectively. To visualise YFP expression to generate the kymographs shown in Fig. S11, epi-fluorescent images were captured at a frame rate of 0.5 frame min^−1^ with an exposure time of 1 s.

In the experiments where we analysed the long-term viability of cells that undergo chemotaxis towards antibiotics (Fig. 3), we used fluid-walled microfluidics^42^. Unlike the BioFlux assays, where cells are fully surrounded by PDMS and glass walls, these open microfluidic devices allowed us to reconfigure channels mid-experiment to isolate and extract cells as they undergo chemotaxis. These experimental methods are explained in detail in a companion publication^43^. Fluid-walled channels were fabricated using Dulbecco’s Modified Eagle Medium (DMEM) + 10% Fetal Bovine Serum (FBS) on untreated, 50 mm glass-bottomed culture dishes (MatTek) using a custom-designed ‘printer’ (iotaSciences Ltd, Oxford, UK). Interfacial forces pin the infused media to the glass surface, which is then overlaid with the fluorocarbon FC40 (iotaSciences Ltd), an immiscible and bio-compatible liquid that prevents the evaporation of the fluid within the channels. TB growth medium was then infused through the device to replace the DMEM + FBS used for fabrication^42^.

Antibiotic gradients were generated using ‘m-shaped’ devices where the flow from two inlet arms merges together in a central channel (Fig. S18; see also^43^). Using the printer, a 1:1 co-culture of YFP-labelled WT and unlabelled *ΔpilG* cells was infused into the device at 0.4 µl min^−1^ and the cells were then left to attach to the bottom glass surface of the channel for 10 min in the absence of flow. The device was then transferred to the microscope, and growth medium was infused into the two inlets using 25 G needles at a rate of 0.1 µl min^−1^ per inlet. The needles were connected to 500 µl glass syringes (Hamilton) that were mounted on separate syringe pumps (PhD Ultra, Harvard Apparatus) using 1 m of tubing (PTFE, Adhesive Dispensing Ltd). Ciprofloxacin (100X MIC) was added to one of the syringes to generate a steady gradient in the channel^43^. The growth medium in both syringes was supplemented with 10% spent medium in order to elicit the chemotactic response more rapidly (see Fig. 2B). To ensure the flow was steady over the course of the experiment, fluid was withdrawn at the same rate it was injected (0.2 µl min^−1^) from a circular sink at the end of the test channel using a third syringe (Plastipak, 10 mL, Becton Dickinson) mounted on a third syringe pump (PhD Ultra, Harvard Apparatus). Cells were imaged using brightfield microscopy immediately downstream of the junction between the two inlet arms at a rate of 1 frame min^−1^. Epifluorescence imaging was used to compare the distribution of unlabelled *ΔpilG* and YFP-labelled WT cells at the beginning and end of the experiment with an exposure time of 150 ms.

After a sufficient number of cells had migrated towards ciprofloxacin (≈24 h), we re-configured the central channel of the device using a PTFE (polytetrafluoroethylene) stylus^44^ mounted to the microscope condenser to isolate different populations of migrating cells within a separate fluid chamber (Fig. 3A–D; see^43^ for details). Immediately prior to this re-configuration, we reduced the concentration of ciprofloxacin flowing through one of the inlet arms from 100X MIC to 3X MIC using a fourth syringe (Plastipak, 1 mL, Becton Dickinson) mounted on a separate syringe pump (PhD Ultra, Harvard Apparatus). During this process, there was a ≈ 20 s period during which flow passed through the 100X MIC and the 3X MIC syringes simultaneously and this shifted the antibiotic gradient towards the antibiotic-free side of the channel. In this period, any cells that had only recently initiated chemotaxis would therefore experience a very brief increase in background antibiotic concentration.

We also expect that the channel reconfiguration process might generate lateral flows that could potentially transport a small number of antibiotic naïve-cells into the isolated fluid chamber. To ensure that none of these antibiotic naïve-cells were able to grow, cells within the fluid chambers were exposed to 3X MIC ciprofloxacin for 2 h. In separate experiments that exposed antibiotic-naive cells to 3X MIC ciprofloxacin in liquid culture, fewer than 1 in 10^5^ cells survived after 2 h. Even in the experiment where the channel was reconfigured at Position A (the position for which the largest number of cells were isolated, Fig. 3A), we estimated that there were only approximately 1000 cells in the fluid chamber. Therefore, even if all 1000 cells in the chamber were antibiotic-naive, we would expect that 99% of the time all cells would be killed by the subsequent treatment. This, along with our finding that only cells isolated from a single experiment were found to be viable, suggests that contamination of the isolated fluid chambers with antibiotic naïve-cells was not an issue.

After 2 h, the contents of the isolated fluid-chambers were extracted and replaced five times with antibiotic-free growth medium (TB) using the printer, to ensure minimal ciprofloxacin remained within the chambers. During each of the five extractions, the contents were spread onto separate antibiotic-free LB agar (1.5%) plates and monitored for colony growth (Fig. 3F). We also imaged the entire bottom surface of the isolated chamber in brightfield every 30 min to monitor the viability of the surface-attached cells that remained within the chambers (Fig. 3G). To confirm that all of the antibiotic had been removed from the chambers, ≈25 exponential-phase, antibiotic-naive cells were added at the end of each experiment, which were observed subsequently to undergo rapid cell division, covering the entire bottom surface of the chamber within approximately 24 h (Fig. 3G).

### Study of secretions and other factors produced by cells

To test the effect of diffusible cell products on twitching chemotaxis we followed our previously published protocol^13^. Exponential phase cells were inoculated in 6-well plates in 4 ml of tryptone broth (TB) at a starting optical density (at 600 nm) of 0.25. Cultures in these 6-well plates were then allowed to grow at 22 °C for 20 h under static conditions in order to obtain a dense culture in which cell-produced factors are present at relatively high concentrations^13^. Cultures were centrifuged at 3000 g for 10 min and then filter-sterilised using 0.22 μm filters (Millipore) to obtain cell-free supernatant, which was stored at 4 °C. Fresh cell-free supernatant was prepared on the day of each microfluidic experiment.

To obtain cell-free supernatant from cells growing under flow conditions within our microfluidic channels (Fig. S13), we used the BioFlux 200 system and followed the same protocol outlined above, except that instead of using dual-inlet microfluidic channels, we used single-inlet channels (48-well plate, product-number 910-0004) where 24 experiments can be run in parallel on the same well plate. By comparison, only 8 experiments can be run in parallel with dual-inlet devices. Flowing tryptone broth through 24 channels simultaneously allowed us to collect a relatively large (≈4 ml) volume of spent growth medium from the outlet wells at the end of the 16 h long experiment. As described above for cultures growing under static conditions, this spent medium was then centrifuged at 3000 *g* for 10 min and filter-sterilised using 0.22 μm filters (Millipore) to obtain cell-free supernatant.

### Image analysis and calculations of movement bias

All images were processed using the open-source software Fiji^56^ and its associated plugins, and all tracking data were exported from Fiji to Matlab (Mathworks) for subsequent analysis as described previously^23^. Where noted, additional statistical analysis was performed using R.

We quantified chemotaxis by calculating the movement bias (*β*), which is defined as the number of cells moving up the gradient divided by the number of cells moving down the gradient. Because twitching motility is inherently jerky due to the stochastic dynamics of individual pili^57^, we used the direction of a cell’s net displacement over the entire length of its trajectory in our calculation of *β* (with the exception of Fig. 2G and Fig. S17). In addition, we excluded trajectories <1 h long, non-motile cells, cell clusters and cells that do not exhibit appreciable movement from their starting position from these analyses. To do this, we calculated a cell’s net-to-gross displacement ratio (NGDR) – the straight-line distance between a trajectory’s start and end positions divided by the total distance travelled by a cell in that time – and excluded any cells with an NGDR of less than 0.15^23^. However, for the experiments that used 100% cell-free supernatant collected from cells growing under flow conditions (Fig. S13), cells reached high densities relatively quickly and so trajectories were likely to be shorter and more frequently broken by neighbouring cells. To account for these differences, we included all trajectories that were over 15 min in duration and we excluded cells that had an NGDR of less than 0.2. Finally, in all of our analyses, we restricted our measurements of movement bias to cells in the central region of the device, where the chemical gradients were largest (*G* = 1/*C*_MAX_ ∂*C/*∂*y* > 0.001, Fig. 2D, F). While our measurements of the movement bias considered only the motile cells in the central region of our microfluidic device, we note that each of our analyses is typically based on thousands of individual cell trajectories.

While many of our analyses resolve the movement bias as a function of time, the analyses shown in Fig. 2G and S17 required us to bin trajectories both in time and space, with the latter based on the strength of the local antibiotic gradient ∂*C/*∂*y*. The analyses in Fig. 2G and S17, therefore, required more data than our other analyses to obtain a sufficient number of trajectories in each bin. To collect more trajectories of chemotaxing cells in a single experiment, we increased the flow speed by a factor of three, which sharpened the strength of the gradients and allowed strong chemotaxis to be observed over a much larger length of our device (Fig. 2E, F). Using this higher flow speed, we collected data from four consecutive fields of view along the length of our device and pooled them together in the same analysis. In comparison, the other experiments that used the lower flow speed only used cell trajectories collected in a single field of view that was positioned directly downstream of the divider between the two inlets, where ∂*C/*∂*y* is largest. In addition, in Fig. 2G and S17, we calculated the chemotactic bias in a slightly different way compared to our other analyses, as there is a possibility that a trajectory could move between spatial bins (illustrated in Fig. 2C, E) over its length. Therefore, in Fig. 2G and S17 we calculated the instantaneous cell movement bias based on the direction of trajectories at each individual timepoint. The analyses in Fig. 2C–G and S17, therefore were not able to average out the irregular, jerky movement that is characteristic of pili-based motility in the same way as in our other analyses, which thus generated slightly smaller movement bias values.

To quantify how the number of cells attached to the surface changes over time in our isolated fluid chambers (Fig. 3G), we used a timeseries of brightfield images collected every 30 min. These images were processed first using the “Normalise Local Contrast” and then the “Subtract Background” plugins in Fiji to remove variations in the intensity of the background. Images were then thresholded to obtain a binary image of the cells. The fraction of the surface covered by cells was then measured by calculating the number of pixels corresponding to cells divided by the total number of pixels.

Brightfield and fluorescent images were processed in a similar manner in order to compare the numbers of YFP-labelled WT and unlabelled *ΔpilG* cells remaining in isolated chambers following channel reconfiguration (Fig. 3E). The total number of cells in a given region was calculated by using our previously outlined cell tracking pipeline to count the number of cells present in our brightfield images^23^. This number was then compared to the number of cells in our fluorescent images to calculate the relative proportion of WT and *ΔpilG* cells, pooling data across four different bio-replicates (total number of cells = 2759).

A similar image processing pipeline was used to quantify how pyocin expression varies across the width of the microfluidic device (Fig. 4D–F). We again detected the pixels corresponding to cells by thresholding brightfield images and then we thresholded the epifluorescence images that quantified pyocin expression. Using these two binary images, we calculated the proportion of pixels within cells that also corresponded to appreciable pyocin expression. This quantity estimates the relative proportion of cells that are expressing pyocin and was binned in rows of 10 pixels along *y* to calculate how pyocin expression varies across the width of the device. To reduce the effect of solitary cells, we smoothed this proportion with a moving average filter.

We used previously described methods^23^ to both automatically detect when twitching cells reverse direction and quantify how the reversal rate differs for cells travelling up the gradient versus down the gradient (Fig. S9). Reversals are relatively rare events (cells reverse direction approximately once every several hours) so a relatively large number of cells must be tracked to obtain reliable measurements of the reversal rate. Cells were imaged at a frame rate of 1 min for ≈10 h and at each time point, a cell can either carry on moving in a relatively straight line or reverse direction. Given our large sample size and the low probability that a cell reverses direction at any given time point, we assumed that reversals can be modelled using a Poisson distribution. We used this assumption to perform an exact Poisson test (using the ‘poisson.test’ function in R) to confirm whether the difference in reversal rates between cells moving up and down the gradient was significant (Fig. S9). We also used this assumption to calculate the confidence intervals of our experimentally measured reversal rates.

The rose plots shown in Fig. 1 show histograms of the overall trajectory movement direction, measured from a trajectory’s origin to its terminus (calculated using the ‘ksdensity’ function in Matlab, MathWorks), for all trajectories longer than 10 min.

The kymographs shown in Fig. S11 were obtained by imaging either WT or *pilG* mutant cells that constitutively express yellow fluorescent protein (see^23^). Epi-fluorescent images were captured every 2 min and for each frame, the fluorescent intensity (*F*) was averaged in *x*. Plotting the logarithm of *F* allows us to visualize both the fainter signals produced by individual cell movement (visible as light blue streaks) and the much stronger signals produced by densely packed biofilm (visible as larger red patches).

### Statistics and reproducibility

For most of our experiments (Figs. 1, 2, 4 and S2, S4–S6, S8–S13, S16, S17, S19), the sample size (i.e. the number of cell trajectories) is set by the automated cell-tracking and analysis software outlined above and no statistical method was used to predetermine sample size. No data was excluded from the analyses aside from the trajectories that failed to meet the criteria described above (e.g. the trajectories of non-motile cells). The number of cell trajectories in these experiments is typically very large (*n* > 1000) and these were analysed using standard statistical methods outlined in each figure legend. For each of these experiments, data is shown for two biological repeats. In all other cases, details of statistical analysis and reproducibility are outlined in the figure legends.

### Modelling the distribution of antibiotics and the distribution of a hypothetical antibiotic-induced cell product within the microfluidic device

We used a mathematical model of diffusion to simulate the distribution of antibiotic within our devices (or analogously the distribution of cell products originating upstream of the test section in the absence of an antibiotic). We used this to illustrate how this distribution would differ from that of hypothetical cell products generated in response to antibiotic within the test section of the device (antibiotic-induced cell products).

The distribution of antibiotics in our device is expected to closely follow the distribution of a gradient of fluorescein, which has a similar diffusion coefficient and is readily imaged using confocal fluorescence microscopy (Fig. 1A^23^). Our previous study found that the fluorescein gradient in the BioFlux microfluidic devices can be accurately modelled by solving the time-dependent diffusion equation in one spatial dimension^23^, which has an analytical solution^58^. This model assumes that the flow through the test section of the device moves at a constant speed, so one can estimate how long the two streams of fluid from each inlet have been in contact with one another at each *x* position along the device, allowing the time dependent solution to be transformed into a spatial map of the steady-state concentration field at each [*x*, *y*] position in the device^23^.

We assumed an “initial condition” such that the normalised antibiotic concentration is unity from *y* = −175 µm to 0 and zero from *y* = 0 to 175 µm at *x* = 0. Also, we assumed no-flux boundaries at *y* = −175 and 175 µm, which correspond to the physical boundaries within our device. With these initial and boundary conditions, the solution of the one-dimensional diffusion equation is given by^58^:

1 $$C={C}_{{{{{\rm{MAX}}}}}}\left\{\frac{h}{L}+\frac{2}{\pi }\mathop{\sum }\limits_{n=1}^{{{\infty }}}\frac{1}{n}{{{{{\rm{sin}}}}}} \frac{n\pi h}{L}{{{{{\rm{exp}}}}}} \left(-D{n}^{2}{\pi }^{2}t/{L}^{2}\right){{{{{\rm{cos }}}}}}\frac{n\pi (y+L/2)}{L}\right\}.$$

Assuming constant advection speed, we then transformed this time dependent solution to distance along the device via the substitution *t* = *x U* ^−1^, where *U* = 42.3 µm s^−1^ is the mean flow velocity for the “low flow” and *U* = 127 µm s^−1^ for the “high flow” condition, *L* = 350 µm is the channel width, *h* = *L*/2, and *D* is the diffusion coefficient.

We find that the location of the 1X MIC isocontour predicted by the model shows good agreement with the distribution of biofilm experimentally observed in our microfluidic device for *D* *=* 200 µm^2^ s^−1^, (Fig. 2C, E). However, we also explored different values of *D*, (Fig. S16). This model was also used to group cell trajectories into different bins according to the strength of the antibiotic gradient they have been exposed to (Fig. 2G and Fig. S17). Here, the flow rate is 1.1 × 10^6^ µm^3^ s^−1^ (“low flow speed”) or 3.3 × 10^6^ µm^3^ s^−1^ (“high flow speed”) and the diffusion coefficient is assumed to be 200 µm^2^ s^−1^.

We used a similar modelling framework to simulate the distribution of a hypothetical cell product produced only in the presence of the antibiotic. In the test section of our dual-inlet microfluidic experiments, cells exposed to sub-MIC concentrations of antibiotic formed biofilms (Fig. 1A, 2C, E), where they could hypothetically generate compounds in response to the antibiotic. Intuitively, one would expect that the concentration of such a compound would increase as fluid moves in the downstream direction, as it passes over the top of more and more antibiotic-exposed cells. To model this scenario, we again used a time-dependent, one-dimensional model of diffusion, assuming that the concentration of any antibiotic-induced cell product is zero at *x* = 0 (the “initial condition”). This assumption is supported by our observation that cells upstream of the test section are rapidly cleared from the arm of the device containing antibiotics, suggesting that the amount of antibiotic-induced cell products originating upstream of the test section was negligible.

Whilst it is difficult to speculate how cells exposed to different concentrations of antibiotic might differ in the rate at which they produce an antibiotic-induced product, for simplicity, we assumed that cells on the top half of the device generate this compound at a constant rate per unit area. More specifically, we included a source term in our model of diffusion that is equal to unity from *y* = 0 to 175 µm and zero for *y* = −175–0 µm. We numerically integrated the governing diffusion equation using the “pdepe” function in Matlab (Mathworks). For consistency, we assumed that the diffusion coefficient of the hypothetical antibiotic-induced cell product is the same as that of the antibiotic (200 µm^2^ s^−1^), but the general trends illustrated in Fig. S15 do not depend on the exact value of *D*.

The distribution of antibiotic and the distribution of the hypothetical antibiotic-induced cell product show starkly different patterns. The strength of the antibiotic gradient is maximum when the fluid from the two inlets first meet one another at *x* = 0 and rapidly decreases further downstream. In contrast, the strength of the hypothetical antibiotic-induced cell product is zero at *x* = 0 and increases steadily as one moves downstream (Fig. S15). Our massively parallel cell tracking analyses show that the movement bias is predicted by the former distribution (Fig. 2G and Fig. S17), suggesting that the directed movement of cells in our experiments is not driven by a cell product induced by a sub-MIC concentration of antibiotics.

### Reporting summary

Further information on research design is available in the Nature Portfolio Reporting Summary linked to this article.

## Supplementary information

Supplementary Information

Description of Additional Supplementary Files

Movie 1

Movie 2

Movie 3

Movie 4

Movie 5

Reporting Summary

## Data Availability

The full data set that supports the findings of this study are available from the corresponding authors upon request. Source data for Figs. 1–4 and S1, 2, 4–6, 8-10, 12, 13, 15, 17 and 19 are provided with this paper. Source data are provided with this paper.

## Source data

Source data

## Code availability

All of the code used to generate the findings of this study is available from the corresponding authors upon request.
