# Supplementary material for: Suicidal chemotaxis in bacteria
Source: Nat Commun. 2022 Dec 9;13:7608. doi: 10.1038/s41467-022-35311-4 (PMC9734745; doi:10.1038/s41467-022-35311-4)
Supplement: Supplementary file 1 — Supplementary Information [file 41467_2022_35311_MOESM1_ESM.pdf]

Supplementary Information for:

Suicidal chemotaxis in bacteria

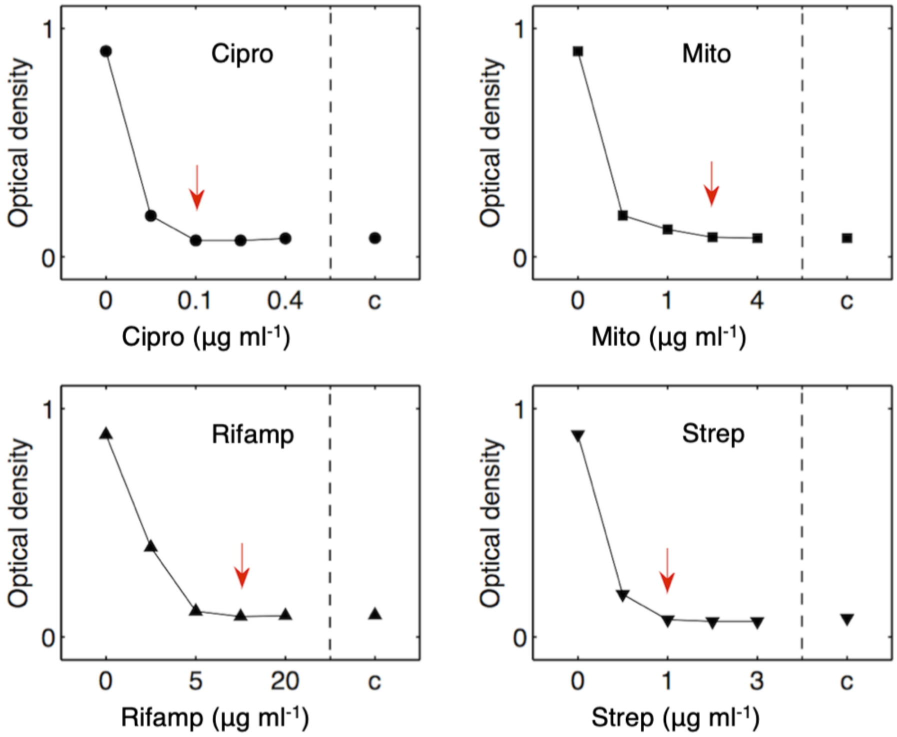

Figure S1. Quantifying the minimum inhibitory concentration (MIC) of different antibiotics in shaken culture. Here we show the optical density after 20 h in different concentrations of 'Cipro' = ciprofloxacin, 'Mito' = Mitomycin C, 'Rifamp' = Rifampicin, and 'Strep' = Streptomycin. 'c' denotes controls without cells conducted in pure tryptone broth. Red arrows denote the minimum inhibitory concentration (MIC) for each antibiotic, which is defined here as the minimum concentration of antibiotic that prevents detectable growth after 20 h. Error bars representing standard error centered on the mean of eight independent bio-replicates are too small to see and fall within each marker shown. Source data are provided as a Source Data file.

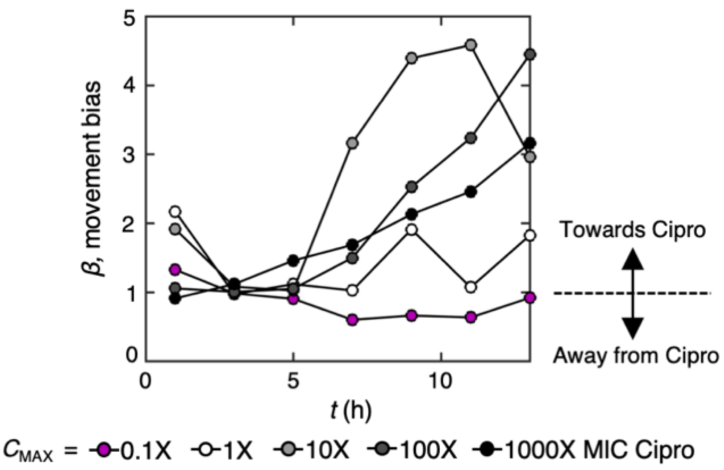

Figure S2. Cells bias their motility towards harmful antibiotic concentrations. The data shown here come from a biological repeat of the experiments shown in Fig. 1D, where cells were exposed to ciprofloxacin gradients across a range of concentrations. After an initial delay of between ≈5 to 7.5 h, cells bias their movement towards ciprofloxacin ($\beta > 1$; at $t = 13$ h, a two-sided binomial test rejects the null hypothesis that trajectories are equally likely to be directed towards or away from ciprofloxacin for C_{MAX} = 1X, 10X, 100X and 1000X ($p < 0.0001$ in all cases, n = 384, 411, 483 and 485 cell trajectories respectively), except when ciprofloxacin is used at sub-inhibitory concentrations (C_{MAX} = 0.1X MIC, magenta circles, $\beta \approx 1$; $p = 0.688$, n = 338 cell trajectories). Source data are provided as a Source Data file.

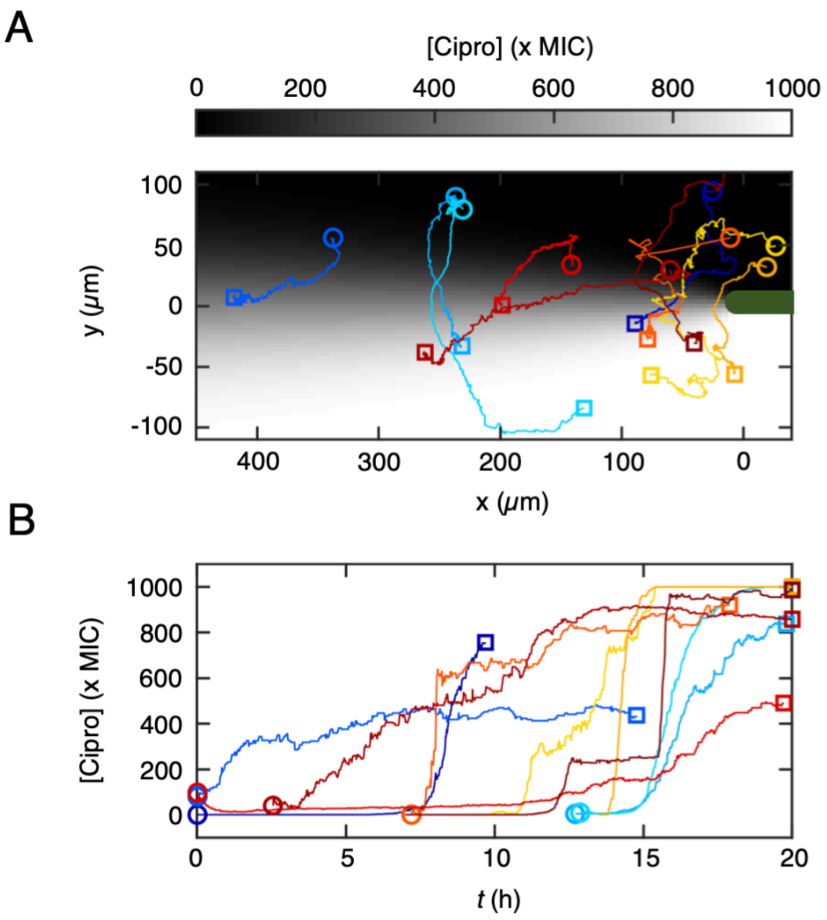

Figure S3. Cells that chemotax towards ciprofloxacin remain motile for hours after reaching antibiotic concentrations corresponding to hundreds of times that of their MIC. (A) While automated tracking can simultaneously follow the motility of hundreds of cells, cell trajectories periodically break when two cells come into close proximity and touch one another. To illustrate cell movement over long periods of time in the densely crowded conditions found in our experiments, we therefore used manual cell tracking to follow the movement of 10 representative cells moving in a ciprofloxacin gradient that varies from a concentration of zero to 1000 times the MIC. **(B)** We used the mathematical model of ciprofloxacin distribution within our device (Methods) to resolve the concentration that these cells experienced over time. This shows that cells are capable of moving into concentrations hundreds of times that of the MIC and remain motile for hours, even though they are ultimately non-viable (Fig. 3).

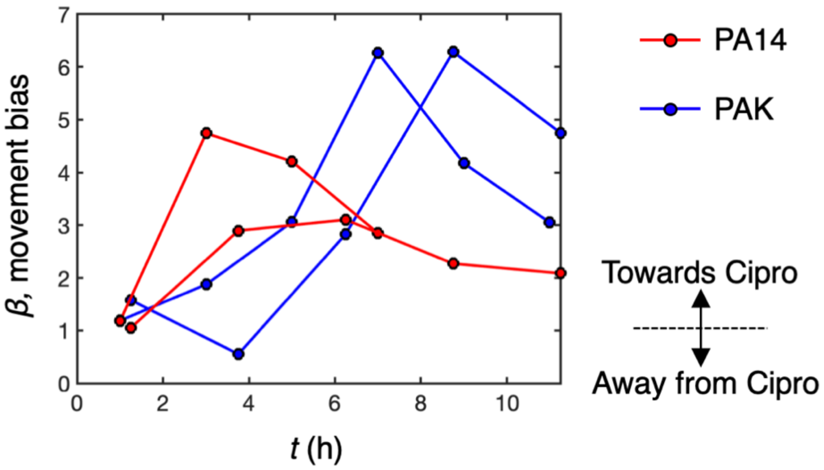

Figure S4. Chemotaxis towards antibiotics is also observed in strains PA14 and PAK. The focal strain of *P. aeruginosa* used throughout this study is PAO1 but we also find that strains PA14 (red lines) and PAK (blue lines) bias their twitching motility towards increasing ciprofloxacin concentrations when exposed to ciprofloxacin gradients (C_{MAX} = 10X MIC, $\beta > 1$; at t = 11 h, a two-sided binomial test rejects the null hypothesis that trajectories are equally likely to be directed towards or away from ciprofloxacin with $p < 0.0001$ in all cases; n = 587, 540, 638 and 653 cell trajectories for the two PA14 and PAK biological repeats respectively). Note that spent medium collected from PA14 and PAK liquid cultures respectively was added to both sides of the gradient at a concentration of 10% because this was found to promote chemotaxis in experiments with PAO1 (see Methods). In both cases, data are shown for two biological repeats. See also Movie 2. Source data are provided as a Source Data file.

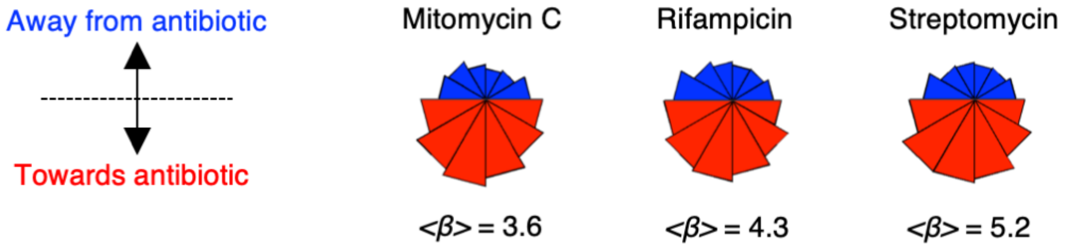

Figure S5. Cells bias their motility towards a range of different antibiotics. Data shown here come from a biological repeat of the experiments shown in Fig. 1E. In both cases, circular histograms of the direction of cell motility (number of trajectories from left to right, n = 3968, 2655 and 6984) and measurements of $<\beta>$ demonstrate that, in addition to ciprofloxacin, cells bias their movement towards mitomycin C, rifampicin and streptomycin (C_{MAX} = 10X MIC in all cases). Red segments show cells moving towards increasing antibiotic concentrations, whilst blue segments show cells moving towards decreasing concentrations. In all three cases, a two-sided binomial test rejects the null hypothesis that trajectories are equally likely to be directed towards and away from increasing antibiotic concentrations ($p<0.0001$, n = 3968, 2655, and 6984 cell trajectory points for mitomycin C, rifampicin and streptomycin gradients respectively). Source data are provided as a Source Data file.

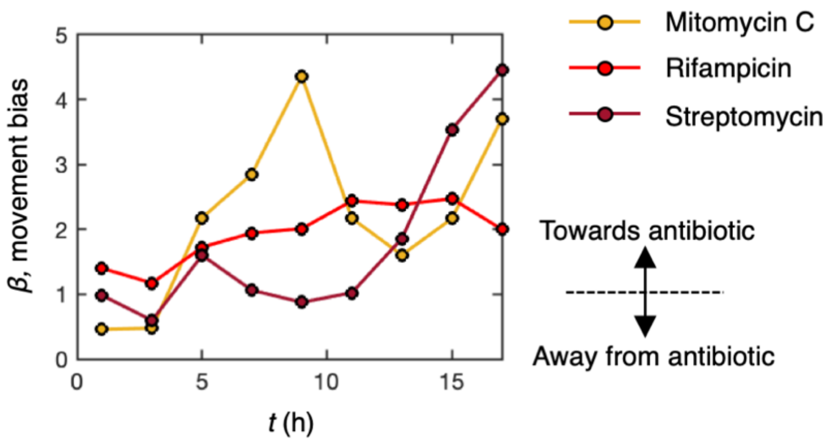

Figure S6. Temporal trends in cell movement towards antibiotics. In addition to ciprofloxacin, cells were also exposed to gradients of mitomycin C, rifampicin and streptomycin (see data shown in Fig. 1E). After a period of unbiased movement ($\beta \approx 1$), cells begin to bias their movement ($\beta > 1$) towards these three antibiotics ($t \gtrsim 5$ h for mitomycin and rifampicin and $t \gtrsim 12.5$ h for streptomycin; at $t = 17$ h, a two-sided binomial test rejects the null hypothesis that trajectories are equally likely to be directed towards or away from antibiotics with $p < 0.0001$ in all cases, $n = 206$, 1005, and 581 cell trajectories for mitomycin C, rifampicin and streptomycin gradients respectively). Source data are provided as a Source Data file.

A

B

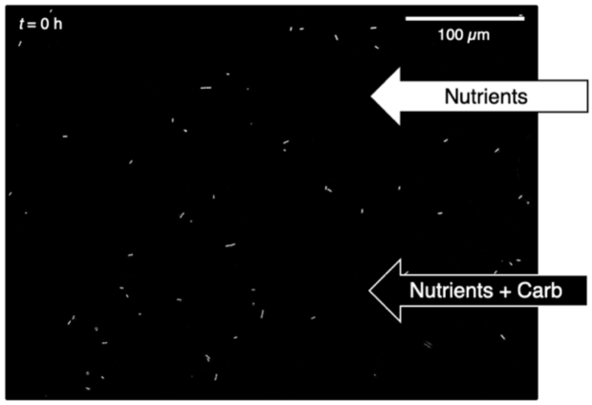

Figure S7. Carbenicillin causes extreme cell elongation, preventing cell motility. (A, B) We exposed cells to a gradient of carbenicillin *(*C_{MAX} = 10X MIC) to test whether *P. aeruginosa* also undergoes chemotaxis towards *β* -lactam antibiotics. After 5 h exposure to carbenicillin, cells become highly elongated and lack the ability to move. Images shown are representative of two independent bio-replicates.

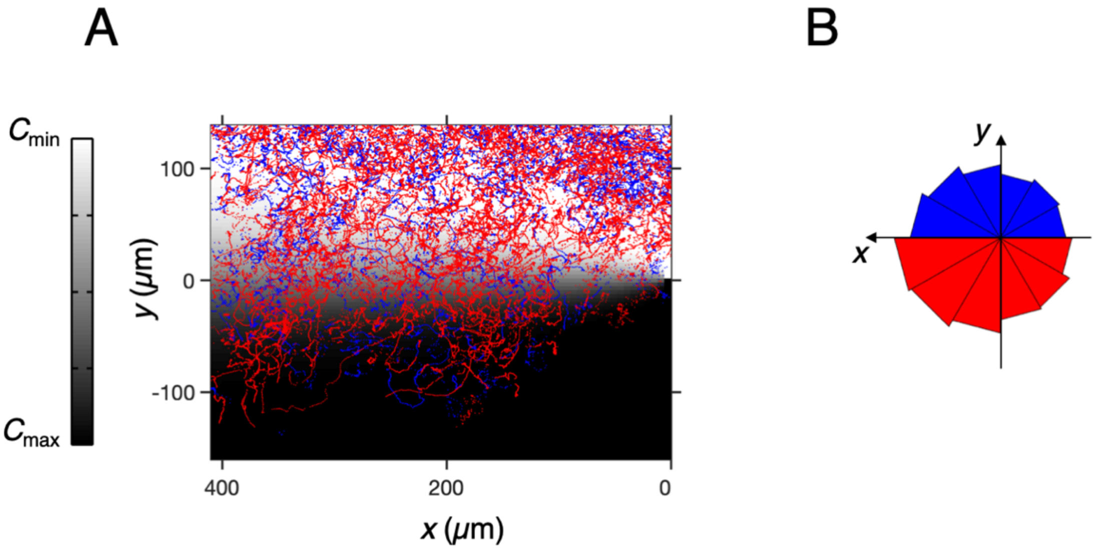

Figure S8. Cells bias their motility towards the antimicrobial agent hydrogen peroxide. (A) Cells were exposed to a gradient in hydrogen peroxide concentration (shown in grayscale, C_{MAX} = 1 mM). Cell trajectories are overlaid atop the gradient with red and blue trajectories moving towards increasing and decreasing concentrations respectively. **(B)** A polar histogram containing trajectories pooled across the two biological repeats shown in panels A and B (*n* = 7326) reveals that cells are more likely to move towards higher hydrogen peroxide concentrations (red segments) rather than towards lower concentrations (blue segments). A two-sided binomial test rejects the null hypothesis that trajectories are equally likely to be directed towards and away from increasing hydrogen peroxide concentrations (p<0.0001, n = 7326 cell trajectory points). Source data are provided as a Source Data file.

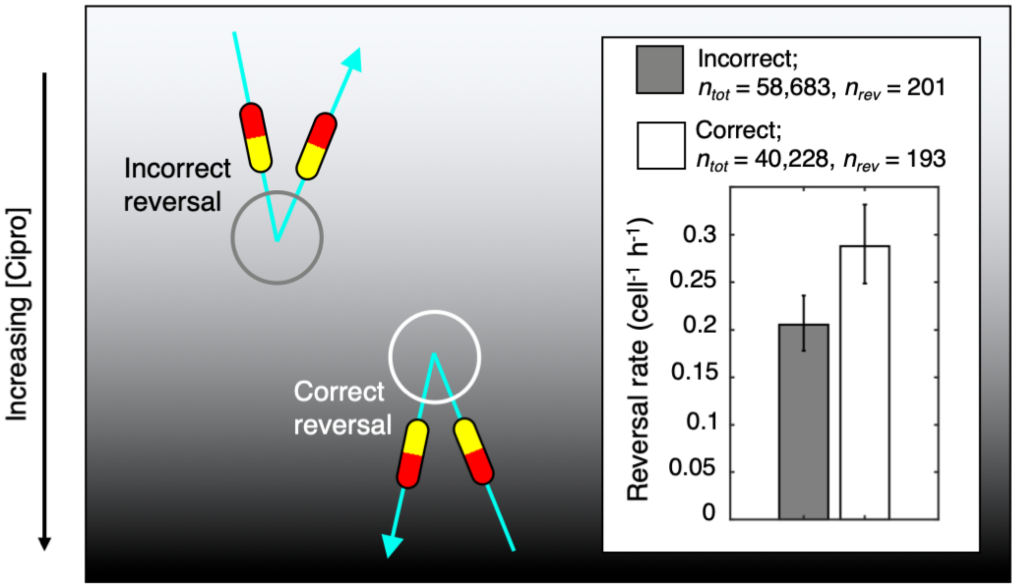

Figure S9. ***P. aeruginosa*** **biases its motility towards ciprofloxacin by actively reversing direction.** Twitching *P. aeruginosa* cells have the ability to actively regulate the direction of their movement by reversing direction approximately 180° (1,2). In the context of a chemoattractant gradient, these reversals can either be "correct" (white circle), which reorient a cell previously moving away from the chemoattractant, or "incorrect" (grey circle), which do the opposite. Whilst reversals are relatively rare (i.e. approximately once every five hours), automated cell tracking allows us to follow the movement of thousands of cells and automatically identify when reversals occur (1). In ciprofloxacin gradients, we find that cells are significantly more likely to undergo correct reversals (white bar, inset) than incorrect reversals, which is consistent with previous observations of chemotaxis (1). A two-sided exact Poisson test (Methods) rejected the null hypothesis that the incorrect and correct reversal rate come from the same Poisson distribution (p < 0.001). Data is presented as the mean values +/- 95% confidence intervals, which were calculated assuming that reversals follow a Poisson distribution (Methods). The total number of reversals was n_{rev} = 394 observed across a total of n_{tot} = 98,911 cell trajectory points (equivalent to 1648 hours of cell trajectories). Data shown is representative of two independent bio-replicates. Source data are provided as a Source Data file.

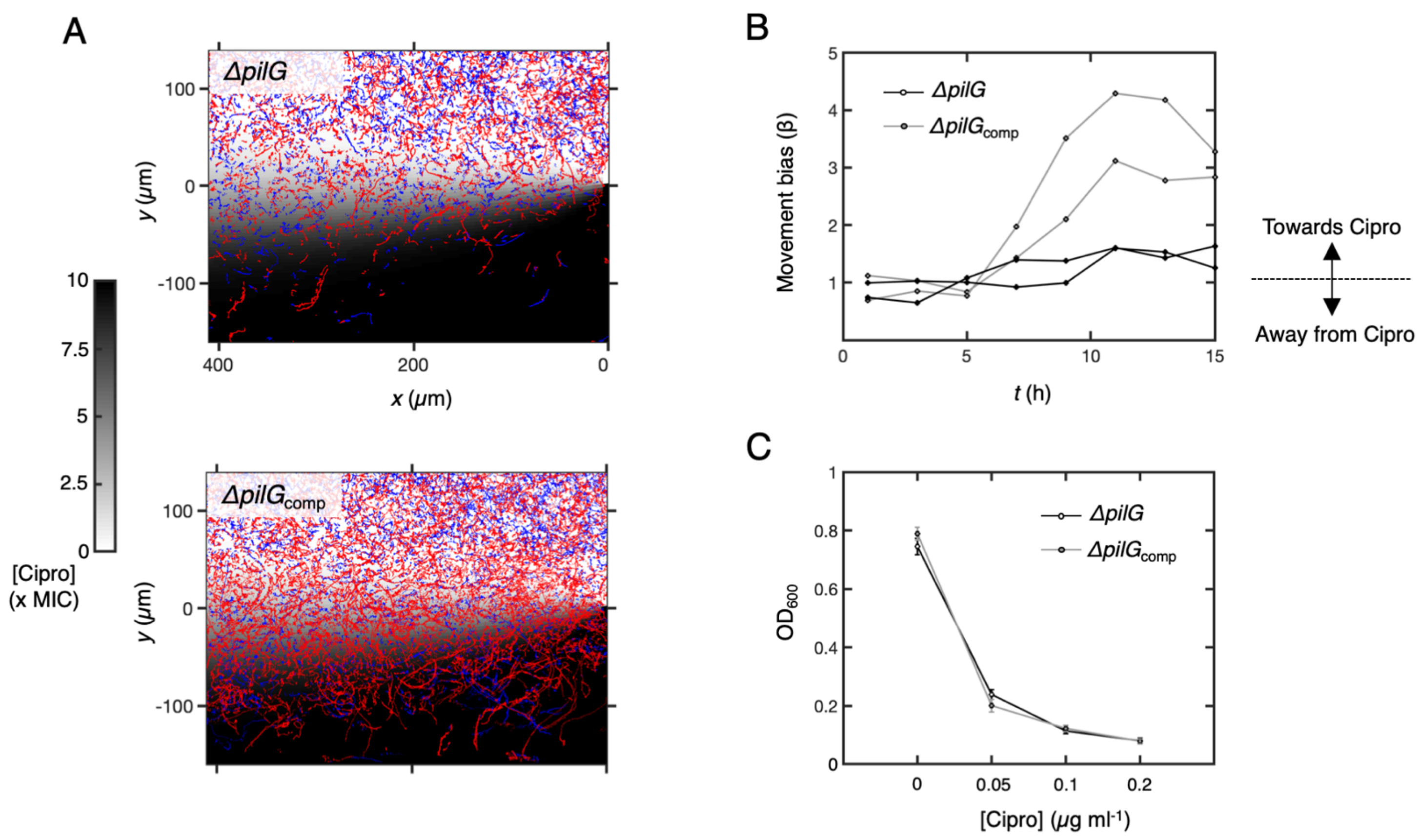

Figure S10. A *ΔpilG* strain that rarely reverses direction has a significantly reduced movement bias towards ciprofloxacin. (A) To test the role of reversals in driving chemotaxis towards antibiotics, we studied a *ΔpilG* mutant that has previously been shown to have a reduced reversal rate and is unable to perform chemotaxis (1,3). We exposed both the mutant strain (*ΔpilG*) and its complement (*ΔpilG*$_{comp}$) to a gradient of ciprofloxacin (C_{MAX} = 10X MIC ciprofloxacin; see also Movie 3). Cell trajectories are colour coded according to whether they move towards increasing (red) or decreasing (blue) ciprofloxacin concentrations. **(B)** Whilst the movement bias β of *ΔpilG* cells remains close to 1 throughout the experiment ($\beta \approx 1$), the *ΔpilG*$_{comp}$ strain clearly begins to move towards increasing ciprofloxacin after ≈7.5 h ($\beta > 1$; a two-sided chi-squared test at t = 15 h rejects the null hypothesis that the two strains have equal β values with $p < 0.0001$, n = 3277 cell trajectory points across all datasets). Data are shown for two biological repeats. **(C)** The optical density of *ΔpilG* and *ΔpilG*$_{comp}$ cells in different ciprofloxacin concentrations are nearly the same after an incubation of 20 h in shaken culture, and are similar to previous estimates of PAO1 ((4), Fig. S1), suggesting that PilG does not appreciably affect ciprofloxacin resistance. As noted in the main text, *ΔpilG* cells exhibit reduced motility compared to WT cells in these assays (1) and so our analyses only include cells of each genotype that make appreciable movement from their initial position (Methods). Error bars show standard error around the mean of eight independent bio-replicates. Source data are provided as a Source Data file.

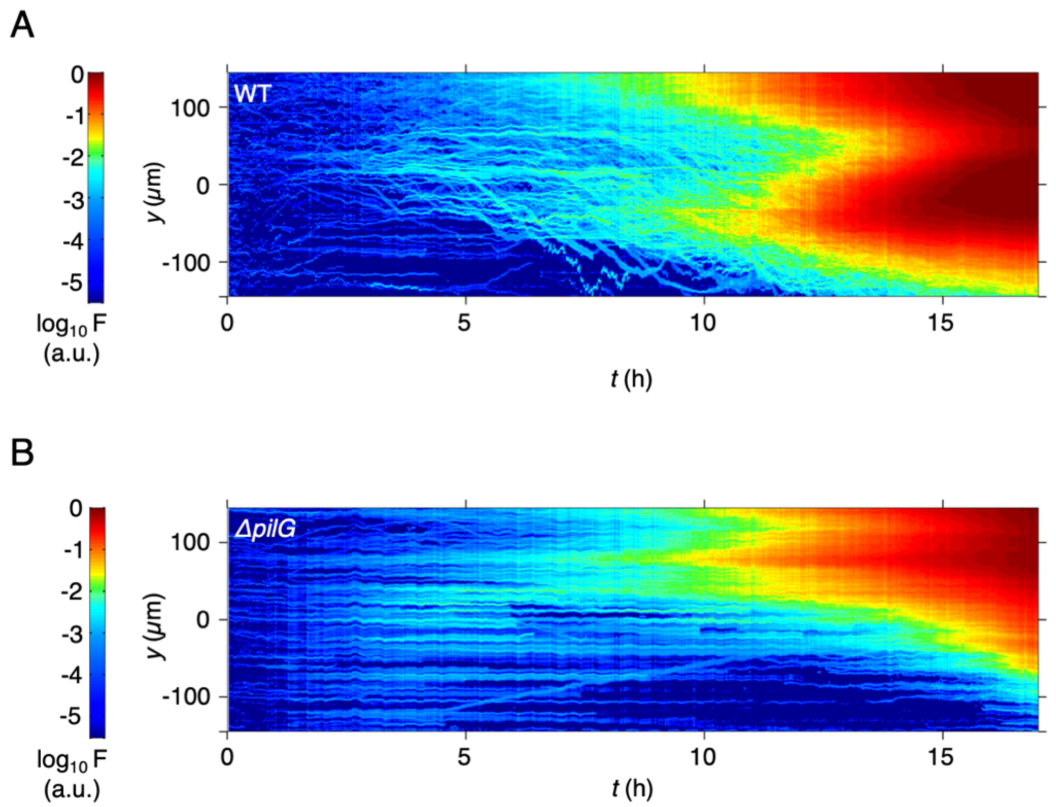

Figure S11. A kymograph of the mean fluorescent intensity of cells that constitutively express YFP as a function of time. We used epi-fluorescent microscopy to image WT cells and mutants lacking PilG in two separate experiments as they responded to a gradient of ciprofloxacin (C_{MAX} = 10X MIC). Here, we averaged the YFP intensity of our images in *x*, (the direction of the flow), allowing us to plot the YFP intensity as a function of both time and distance across the width of the device (Methods). **(A)** We observed that WT cells formed an intense band in the middle of the device after approximately 10 h, which resulted from their chemotaxis towards higher concentrations of ciprofloxacin. **(B)** In contrast, *ΔpilG* cells did not form a separate band in the middle of the device, which is consistent with their impaired chemotaxis (Fig. S10, (1,3)). However, both strains formed thick biofilms in the regions containing sub-MIC concentrations of ciprofloxacin, which appear here as the red bands at the top of both A and B.

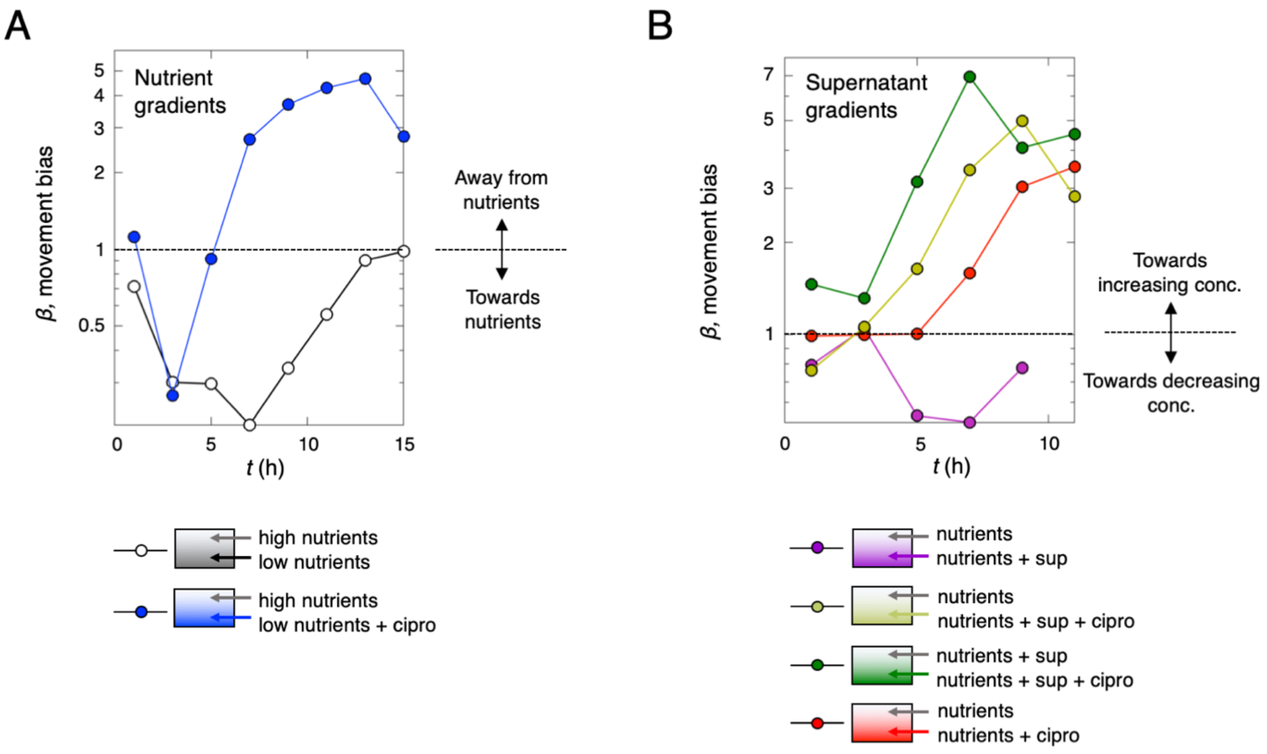

Figure S12. Secondary gradients do not explain movement towards antibiotics. Data shown here come from a biological repeat of the experiments shown in Fig. 2A and B. **(A)** Cells move towards higher concentrations of tryptone (black line, $\beta < 1$; C_{MIN} = 10% to C_{MAX} = 100% of the regular concentration used in our growth medium. A two-sided binomial test at $t = 5$ h rejects the null hypothesis that trajectories are equally likely to be directed towards or away from tryptone ($p < 0.0001$, $n = 98$ cell trajectories). However, if we add ciprofloxacin (C_{MAX} = 10X MIC) to the lower concentration of tryptone, whilst cells initially ($t \lesssim 5$ h) bias their movement towards increasing tryptone concentrations (blue line, $\beta < 1$), this movement is subsequently ($t \gtrsim 5$ h) reversed such that cells then move towards lower tryptone concentrations but increasing ciprofloxacin concentrations (blue line, $\beta > 1$; $p < 0.0001$ and $n = 498$ cell trajectories at $t = 15$ h). **(B)** Cells move away from cell-free supernatant (purple line, $\beta < 1$, C_{MAX} = 10%; a two-sided binomial test at $t = 9$ h rejects the null hypothesis that trajectories are equally likely to be directed towards or away from supernatant ($p = 0.0006$, $n = 656$ cell trajectories) but after a brief (≈5 h) delay this movement bias is reversed when ciprofloxacin (C_{MAX} = 10X MIC) is added to the cell-free supernatant (light-green line, $\beta > 1$; $p < 0.0001$ and $n = 610$ cell trajectories at $t = 11$ h). Cells also move towards increasing ciprofloxacin concentrations when exposed to a background concentration of 10% cell-free supernatant on both sides of the gradient (dark-green line, $p < 0.0001$ and $n = 499$ cell trajectories at $t = 11$ h), here without the initial delay. Source data are provided as a Source Data file.

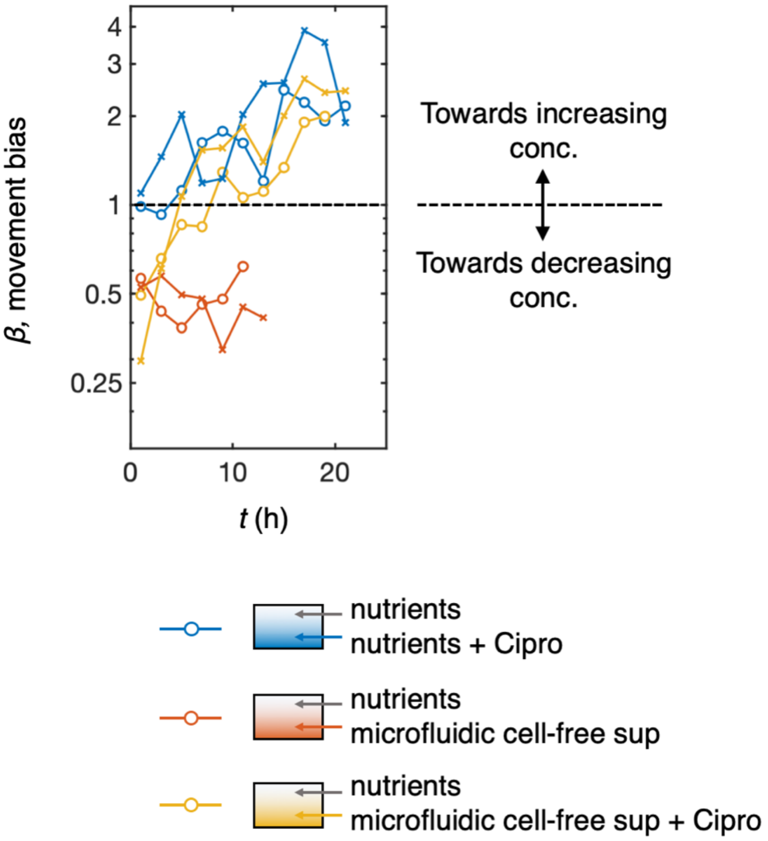

Figure S13. Cells move towards antibiotics despite repulsion from cell-free supernatant collected from microfluidic devices. Cells bias their movement away from 100% cell-free supernatant obtained from microfluidic devices (red lines show two biological repeats, $\beta < 1$; a two-sided binomial test at t = 11 h rejects the null hypothesis that trajectories are equally likely to be directed towards or away from supernatant (p = 0.0016 and 0.0014, n = 89 and 265 cell trajectories)), as they do for cell-free supernatant collected from static-well plates (Fig. 2B). Note that after ≈13 h, the cell density in this gradient, (in which there are no antibiotics present), becomes too high to track cells reliably. If ciprofloxacin (C_{MAX} = 10X MIC) is added to the supernatant (yellow lines), cells initially move away from the antibiotic ($\beta < 1$), but after ≈7 h cells begin moving towards the added ciprofloxacin ($\beta > 1$; $p < 0.01$ and n = 43 and 387 cell trajectories at t = 19 and 21 h in both repeats). A control experiment with a ciprofloxacin gradient only (C_{MAX} = 10X MIC) is shown for comparison (blue lines; $\beta > 1$; $p < 0.001$ and n = 130 and 584 cell trajectories in the two repeats at t = 21 h). See also Movie 4. Source data are provided as a Source Data file.

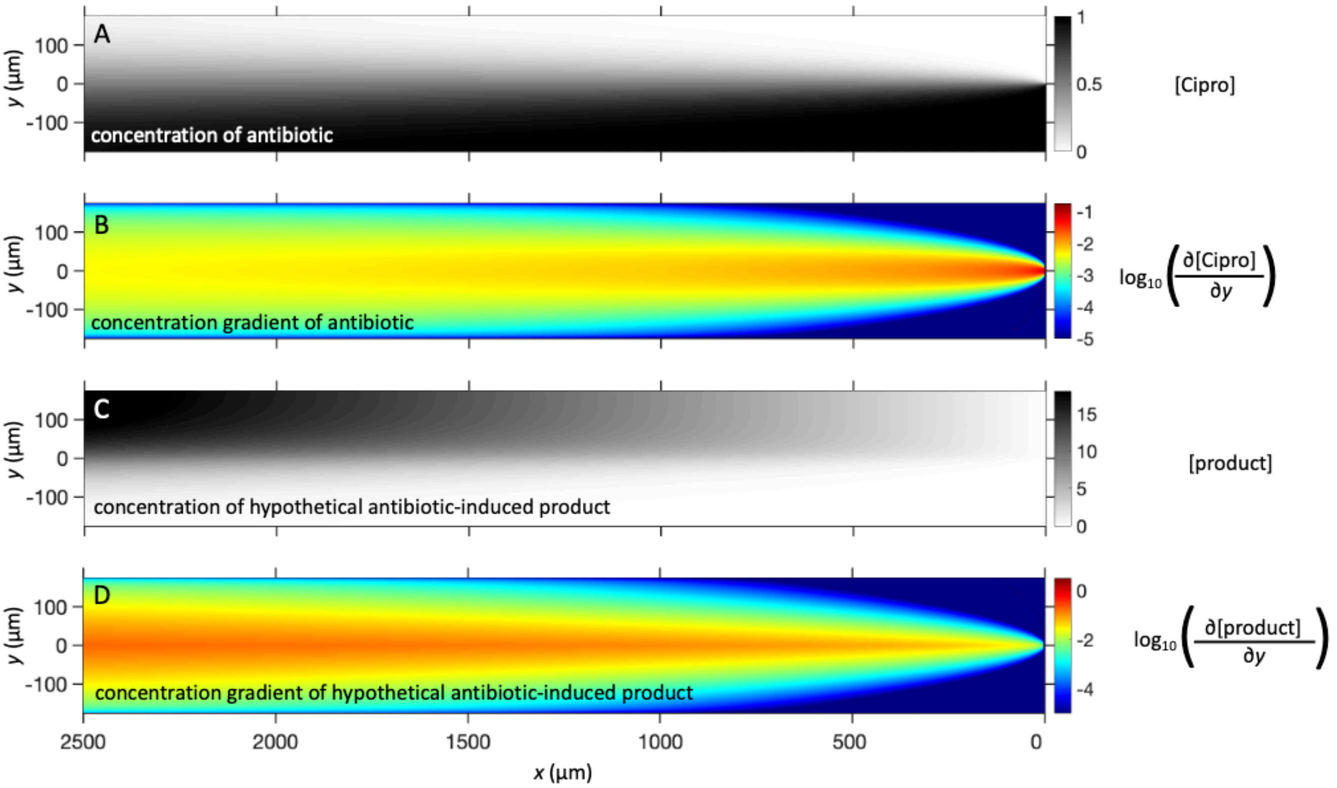

Figure S14. The concentration of a hypothetical cell product released from cells in response to antibiotics would increase in the downstream direction of our device, producing a starkly different distribution compared to that of the antibiotic itself. Here we used a mathematical model of diffusion to simulate both the distribution of antibiotic within the microfluidic device **(A** and **B)**, and the distribution of a hypothetical product that is released by cells in response to the antibiotic, which here is assumed to be produced at a constant rate in the region x = 0 to 175 µm **(C** and **D)**. The concentration and concentration gradients have been normalised and are presented here in arbitrary units. See Methods for further details.

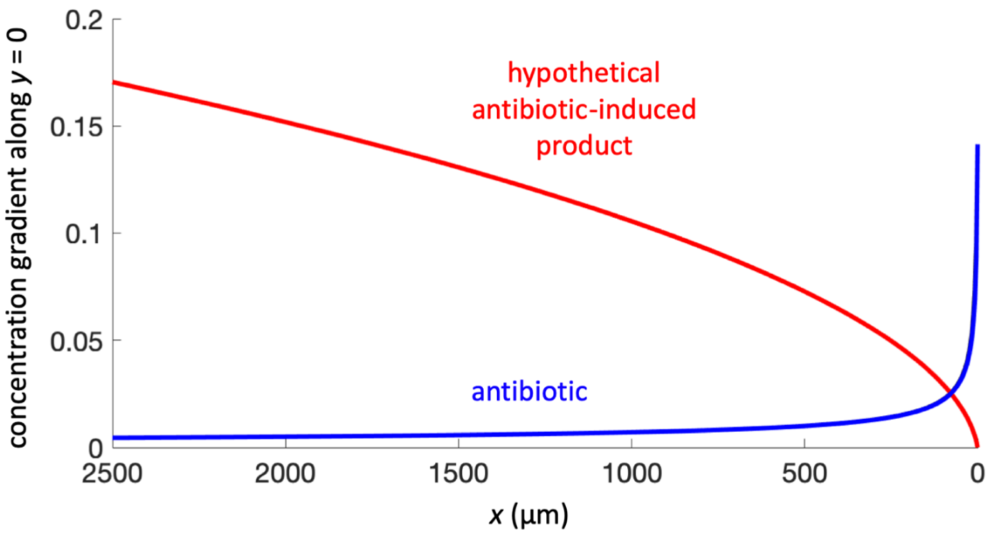

Figure S15. Predictions of chemical gradients in the microfluidic device. The concentration gradient of the antibiotic is predicted to decrease in the downstream direction, whilst the concentration gradient of a hypothetical antibiotic-induced cell product would increase in the downstream direction. The blue and red lines show model predictions of ∂[antibiotic]/ ∂y and ∂[product]/ ∂y, respectively along the centreline of the channel (y = 0, see Fig. S14). Source data are provided as a Source Data file.

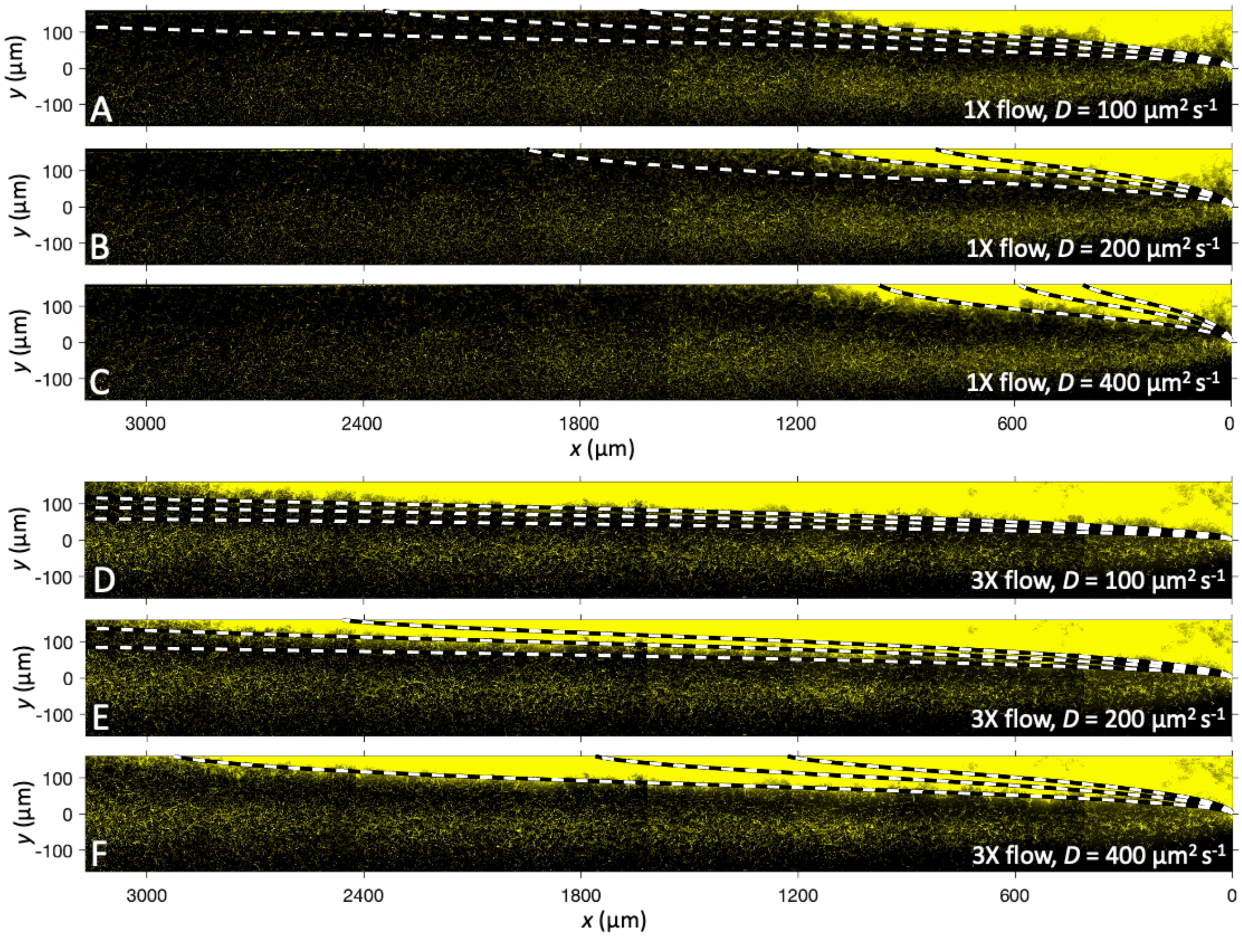

Figure S16. Estimating the antibiotic concentration below which thick biofilms form in our microfluidic devices using a model of diffusion. Here we show experimental images of PAO1 cells that constitutively express YFP in experiments using both our standard flow speed (mean = 42.3 µm sec^{-1}, Panels **A, B, C**) and a three-fold larger flow speed (mean = 127 µm sec^{-1}, Panels **D, E, F**). In each panel, the top dashed-line shows the 0.5X MIC isocontour, the middle dashed-line shows the 1X MIC isocontour, and the bottom dashed-line shows the 2X MIC isocontour. The experimental images shown here are identical to those shown in Fig. 2G and both experiments used a maximum concentration of 1 µg ml^{-1}, which corresponds to 10X the MIC of this strain (Fig. S1). The dashed-lines show modelling results obtained for three different diffusion coefficients, illustrating the model's sensitivity to this parameter. While three previous estimates of the diffusion coefficient of ciprofloxacin vary (5–7), our predictions suggest that the position and shape of the bottom edge of the thick biofilm occurs at a ciprofloxacin concentration of approximately 0.5X to 2X the MIC of this strain.

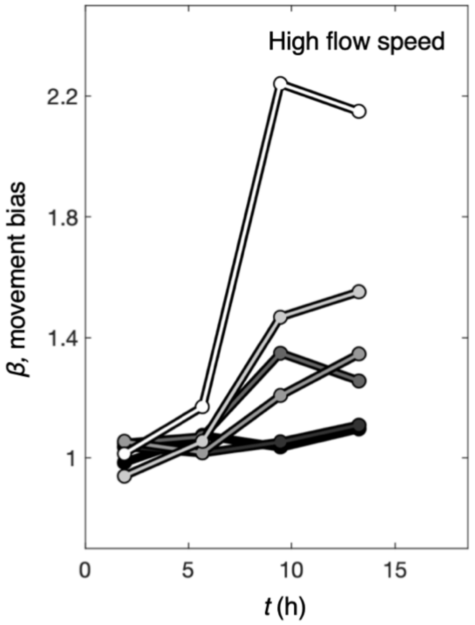

Figure S17. The cell movement bias is largest in regions of the microfluidic device where the antibiotic gradient *∂C/∂y* is strongest. Data shown here come from a biological repeat of the experiments shown in Fig. 2G, where cells were exposed to a ciprofloxacin gradient (C_{MAX} = 10X MIC) using a mean flow speed of 127 µm sec^{-1}, three times that used in our standard assays so that we could quantify strong chemotaxis in four different fields of view simultaneously (see Methods). Here the different lines show the movement bias of cells exposed to the different regions shown in Fig. 2F. Darker shades of grey correspond to regions where the antibiotic gradient ($\partial C/\partial y$) is weaker. A least squares linear regression at t = 13 h of $\log_{10}(\partial C/\partial y)$ against β yielded a slope of 0.320 (95% confidence bounds = 0.0569, 0.584). Source data are provided as a Source Data file.

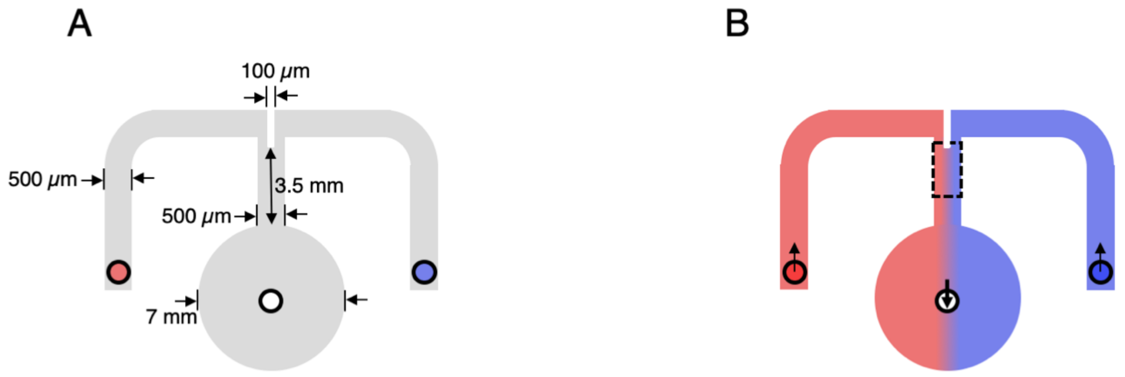

Figure S18. Geometry of fluid-walled microfluidic device used to study long-term cell viability (Fig. 3). (A) We used an m-shaped microfluidic device with two inlet arms feeding into a central channel with a large circular outlet well. A divider was printed at the upstream end of the central channel to prevent upstream mixing of the fluids in the two inlet arms. Inlet tubing was inserted into the ends of the two inlet arms (red and blue circles) and outlet tubing (white circle) was inserted into the middle of the circular outlet. **(B)** To generate antibiotic gradients, nutrient medium (shown in red) was infused through tubing connected to the end of one inlet arm and nutrient medium supplemented with ciprofloxacin (shown in blue) was infused through the other. These two fluids meet in the central arm of the device and flow into the circular outlet, where fluid is withdrawn at twice the rate of infusion through each inlet arm to maintain a constant fluid volume within the device. Diffusion across the width of the central channel generates antibiotic gradients, with the dashed black box showing the approximate position of our imaging window.

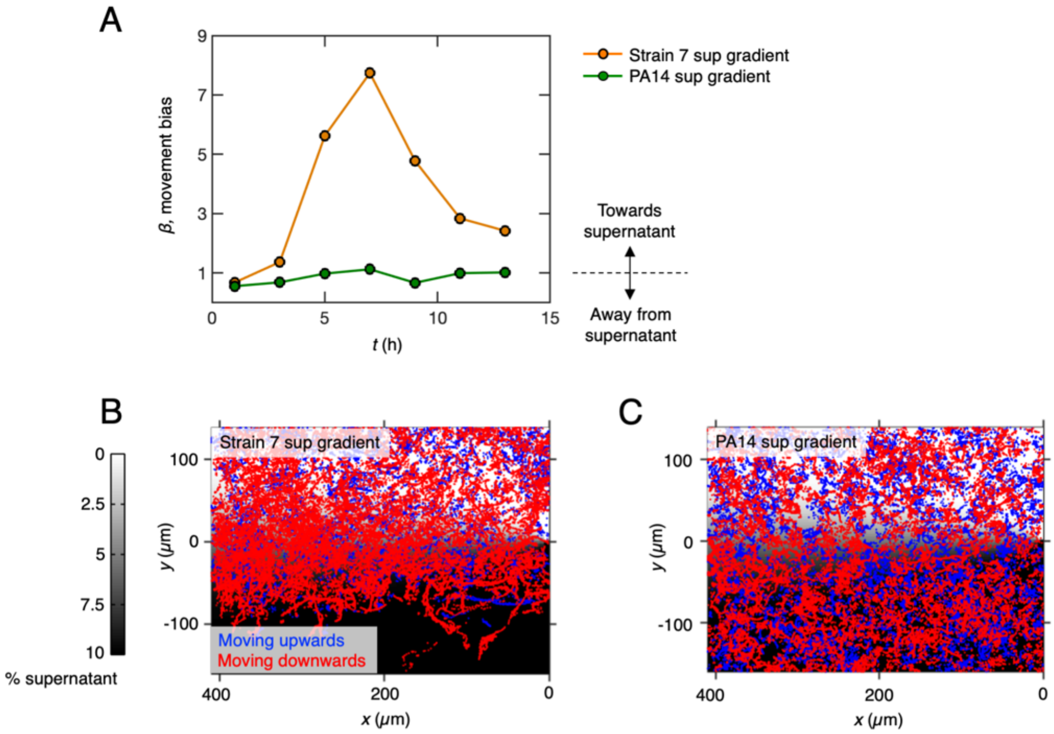

Figure S19. Cells move towards the toxic supernatant of a competitor strain. (A) Data shown here come from a biological repeat of the experiments shown in Fig. 4A. Cells bias their motility towards toxic supernatant collected from Strain 7 ($\beta > 1$, orange line; a two-sided binomial test at $t = 13$ h rejects the null hypothesis that trajectories are equally likely to be directed towards or away from supernatant ($p < 0.0001$, $n = 664$ cell trajectories) but not towards non-inhibitory supernatant collected from PA14 ($\beta \approx 1$, dark-green line; $p = 0.741$ and $n = 471$ cell trajectories at $t = 13$ h). **(B, C)** Cells were exposed to gradients of supernatant concentration (shown in grayscale) collected from either Strain 7 or PA14. Cell trajectories are overlaid atop the gradient with red trajectories showing cells moving towards higher supernatant concentrations and blue trajectories moving towards lower supernatant concentrations. Source data are provided as a Source Data file.

References

1. Oliveira, N. M., Foster, K. R. & Durham, W. M. Single-cell twitching chemotaxis in developing biofilms. *Proc. Natl. Acad. Sci. U. S. A.* **113,** 6532–6537 (2016).
2. Kühn, M. J. *et al.* Mechanotaxis directs Pseudomonas aeruginosa twitching motility. *Proc. Natl. Acad. Sci. U. S. A.* **118,** e2101759118 (2021).
3. Limoli, D. H. *et al.* Interspecies interactions induce exploratory motility in pseudomonas aeruginosa. *Elife* **8,** 1–24 (2019).
4. Brazas, M. D. & Hancock, R. E. W. Ciprofloxacin induction of a susceptibility determinant in Pseudomonas aeruginosa. *Antimicrob. Agents Chemother.* **49,** 3222–3227 (2005).
5. Vrany, J. D., Stewart, P. S. & Suci, P. A. Comparison of recalcitrance to ciprofloxacin and levofloxacin exhibited by Pseudomonas aeruginosa bofilms displaying rapid-transport characteristics. *Antimicrob. Agents Chemother.* **41,** 1352–1358 (1997).
6. Suci, P. A., Mittelman, M. W., Yu, F. P. & Geesey, G. G. Investigation of ciprofloxacin penetration into Pseudomonas aeruginosa biofilms. *Antimicrob. Agents Chemother.* **38,** 2125–2133 (1994).
7. Stewart, P. S. Theoretical aspects of antibiotic diffusion into microbial biofilms. *Antimicrob. Agents Chemother.* **40,** 2517–2522 (1996).
