# Supplementary material for: Suicidal chemotaxis in bacteria
Source: Nat Commun. 2022 Dec 9;13:7608. doi: 10.1038/s41467-022-35311-4 (PMC9734745; doi:10.1038/s41467-022-35311-4)
Supplement: Supplementary file 2 — Description of Additional Supplementary Files [file 41467_2022_35311_MOESM2_ESM.pdf]

Movie 1. Suicidal chemotaxis towards antibiotics. Surface-attached *P. aeruginosa* cells move via twitching motility towards the antibiotic ciprofloxacin (C_{MAX} = 10X MIC), after an initial period of random motility. A static gradient of ciprofloxacin is generated using a dual-inlet microfluidic device with nutrients (tryptone broth) flowing through one inlet and nutrients supplemented with ciprofloxacin through the other. The panel on the right-hand side shows a magnified view of the region marked with the dashed white box in the first frame.

Movie 2. Chemotaxis towards ciprofloxacin is also observed in other strains of *P. aeruginosa*. The left-hand panel shows our focal PAO1 strain, the middle panel shows PA14 and the right-hand panel shows PAK. In all cases, cells were exposed to a static gradient of ciprofloxacin (C_{MAX} = 10X MIC), with nutrients (tryptone broth) flowing through the top-half of the channel and nutrients supplemented with ciprofloxacin flowing through the bottom-half of the channel. Both halves of the channel were also supplemented with 10% cell-free supernatant collected from liquid cultures of each respective strain as this was found to promote chemotaxis towards antibiotics in experiments with PAO1.

Movie 3. A reversal-null *ΔpilG* mutant does not chemotax towards antibiotics. Cells were exposed to a static gradient of ciprofloxacin (C_{MAX} = 10X MIC), with nutrients (tryptone broth) flowing through the top-half of the channel and nutrients supplemented with ciprofloxacin flowing through the bottom-half of the channel. The right-hand panel shows *ΔpilG* mutant cells, which rarely reverse direction and show significantly reduced movement bias towards ciprofloxacin (Fig. S10). In contrast, the left-hand panel shows complemented mutant cells ($\Delta pilG_{comp}$), whose ability to undergo chemotaxis towards ciprofloxacin is restored. Note that *ΔpilG* cells exhibit reduced motility compared to WT cells in these assays and so we only analyse cells that show appreciable movement from their initial position (Methods).

Movie 4. The effects of cell-free supernatant on chemotaxis with and without antibiotics. Left-hand side shows chemotaxis towards antibiotics, where surface-attached *P. aeruginosa* cells move via twitching motility towards ciprofloxacin (C_{MAX} = 10X MIC; see also Movie 1). Middle panel shows cells in a gradient of cell-free supernatant (C_{MAX} = 100% cell free supernatant) extracted from cells grown in tryptone broth within microfluidic devices (‘microfluidic cell-free sup’). Cells are repelled by the cell-free supernatant. Right-hand side shows cells in a combined gradient of cell-free supernatant *and* ciprofloxacin. Cells move towards the ciprofloxacin despite the repulsion of cell-free supernatant (shown in the middle panel).

Movie 5. Cells release bacteriocins (pyocins) during chemotaxis towards antibiotics (ciprofloxacin, C_{MAX} = 10X MIC). Cells turn green when they make bacteriocins as they have a mNeonGreen fluorescent transcriptional reporter for Pyocin R2. Pyocins are released via programmed cell lysis, which can be seen in the movie where it is followed by a transient burst of red colour. This colour is caused by the presence of the dye propidium iodide which binds to the DNA released when cells lyse.
